# Computational Study on the Inhibition Mechanisms of the Ziegler-Natta Catalyst in the Propylene Polymerization Process: Part 1 Effects of Acetylene and Methylacetylene

**DOI:** 10.3390/ijms251910585

**Published:** 2024-10-01

**Authors:** Joaquin Hernandez-Fernandez, Elias Bello-León, Edgar Marquez

**Affiliations:** 1 Chemistry Program, Department of Natural and Exact Sciences, San Pablo Campus, University of Cartagena, Cartagena 30015, Colombia; 2 Chemical Engineering Program, School of Engineering, Universidad Tecnologica de Bolivar, Parque Industrial y Tecnológico Carlos Vélez Pombo, Km 1 Vía Turbaco, Turbaco 130001, Colombia; 3 Department of Natural and Exact Science, Universidad de la Costa, Barranquilla 30300, Colombia; 4 Grupo de Investigación en Ciencias e Ingeniería CECOPAT&A, Chemistry Program, Department of Natural and Exact Sciences, San Pablo Campus, University of Cartagena, Cartagena 131001, Colombia; ebellol@unicartagena.edu.co; 5 Grupo de Investigaciones en Química y Biología, Departamento de Química y Biología, Facultad de Ciencias Básicas, Universidad del Norte, Carrera 51B, Km 5, Vía Puerto Colombia, Barranquilla 081007, Colombia; ebrazon@uninorte.edu.co

**Keywords:** theoretical study, inhibition mechanisms, aliphatic alkynes, propylene polymerization, Ziegler-Natta catalyst

## Abstract

Acetylene and methylacetylene are impurities commonly found in the raw materials used for the production of polymers such as polypropylene and polyethylene. Experimental evidence indicates that both acetylene and methylacetylene can decrease the productivity of the Ziegler-Natta catalyst and alter the properties of the resulting polymer. However, there is still a lack of understanding regarding the mechanisms through which these substances affect this process. Therefore, elucidating these mechanisms is crucial to develop effective solutions to this problem. In this study, the inhibition mechanisms of the Ziegler-Natta catalyst by acetylene and methylacetylene are presented and compared with the incorporation of the first propylene monomer (chain initiation) to elucidate experimental effects. The Density Functional Theory (DFT) method was used, along with the B3LYP-D3 functional and the 6-311++G(d,p) basis set. The recorded adsorption energies were −11.10, −13.99, and −0.31 kcal mol^−1^, while the activation energies were 1.53, 2.83, and 28.36 kcal mol^−1^ for acetylene, methylacetylene, and propylene, respectively. The determined rate constants were 4.68 × 10^11^, 5.29 × 10^11^, and 2.3 × 10^−8^ M^−1^ s^−1^ for acetylene, methylacetylene, and propylene, respectively. Based on these values, it is concluded that inhibition reactions are more feasible than propylene insertion only if an ethylene molecule has not been previously adsorbed, as such an event reinforces propylene adsorption.

## 1. Introduction

Polymers derived from the polymerization of olefins are highly significant products due to their low cost and remarkable, versatile properties, which are applicable across various industrial sectors, including agriculture, pharmacology, food, construction, and more [1,2]. To a large extent, heterogeneous catalysts, such as the TiCl_4_/MgCl_2_ system from the Ziegler-Natta (ZN) catalyst family, are fundamental for the production of high-quality polyolefins. The Ziegler-Natta catalyst consists of a combination of the aforementioned compounds, along with electron donors and a co-catalyst, such as trimethylaluminum (AlEt_3_) [3,4]. This catalytic system has been the subject of numerous computational [4,5,6,7] and experimental [8,9,10] studies, which have investigated its behavior under various conditions, especially about TiCl_4_/MgCl_2_ [8,9,10].

The historical trajectory of the Ziegler-Natta catalyst has represented a milestone of monumental importance in the technology of stereoregular polymer synthesis, both academically and industrially, since the 1950s. The discovery of this catalytic system, conducted by Karl Ziegler in 1953 and Giulio Natta in 1954, marked a turning point in the dominance of stereoregular polymers of natural origin. The fourth generation of Ziegler-Natta catalysts, with MgCl_2_ as the support, introduced a revolutionary improvement in the properties of produced polyolefins, spurring research on the stereospecific polymerization of alpha–olefin and diene monomers. This advancement has enabled the synthesis of new polyolefin materials [7,11,12,13].

In 1995, Huang and Rempel confirmed the identification of the Cp_2_MR^+^ complex as the active species in Ziegler-Natta catalysts, providing clarity regarding the stereospecificity and structure of the active site. Their research also highlighted the influence of substitution patterns on the cyclopentadienyl (Cp) ligand, which enabled the synthesis of polymers with specific properties, enriching polymerization catalysis and underscoring the commercial potential of metallocene catalysts in the polyolefin industry, ensuring their future relevance. Cationic alkyl metallocene complexes, represented by the formula Cp_2_MR^+^, emerged as crucial components in polymerization catalysis. These complexes were obtained in the absence of methylaluminoxane (MAO) by reacting dialkyl metallocenes, such as Cp_2_Zr(CH_3_)_2_, with strong Brønsted acid salts in a 1:1 molar ratio. The resulting active species was a 16-electron cationic complex, typically in the form [Cp_2_Zr(CH_3_)_2_]^+^, whose reactivity was significantly influenced by the counterion present in the system. The relevance of Cp_2_MR^+^ complexes lay in their ability to facilitate polymerization catalysis, especially under conditions where the absence of MAO and the presence of specific counterions were critical to avoid competitive coordination with the olefin monomer at the metal coordination site. These findings underscored the importance of understanding counterion interactions and the electronic structure of cationic alkyl metallocene complexes to optimize their performance in catalytic applications [11]. In 1997, Soga and Shiono made significant advancements in catalysis, including the development of efficient catalysts for the polymerization of propene, the investigation of the effect of metal chlorides on polymerization rate and the electronic structure of active metal ions, as well as the understanding of gnostic interactions. Additionally, they improved catalytic activity by milling TiCl_3_ and created additive-free catalysts to produce isotactic polypropylene. These findings were fundamental for the advancement in catalysis [14]. In 2006, Chang et al. reported on a unique “sea urchin” crystal structure in a fourth-generation Ziegler-Natta catalyst used in propylene polymerization. These discoveries suggest different morphologies of MgCl_2_ crystals, which can impact activity and selectivity in polymer production [15]. In 2007, Andoni et al. presented a new model of the Ziegler-Natta catalyst for ethylene polymerization. Through detailed preparation and characterization, they revealed changes after treatment with TiCl_4_, including the formation of voids in the MgCl_2_·nEtOH film [16]. In 2015, Koen et al. highlighted the utility of solid-state nuclear magnetic resonance (SSNMR) in the characterization of Ziegler-Natta and metallocene catalysts. They emphasized the sensitivity of nuclei such as 35Cl, 47,49Ti, and 91Zr, underscoring their value in investigating catalytically active materials. These findings emphasize the importance of SSNMR in understanding the mechanisms of these catalysts [17]. All these studies and others have contributed to the progressive development and advancement of generations of Ziegler-Natta catalysts (TiCl_4_/MgCl_2_), resulting in undeniable improvements in terms of process optimization, reaction yield, and understanding of mechanisms and influencing factors (see Table 1). These aspects result in the increased effectiveness of the Ziegler-Natta catalytic system. However, this same effectiveness also makes the catalysts more vulnerable to attack by unwanted molecules present in the environment that are not part of the catalysis process. These molecules, considered impurities, can negatively affect the catalysis process by acting as inhibitors [18].

A significant portion of the raw materials used in the production of polypropylene and polyethylene, such as propylene and ethylene, respectively, is derived from petroleum, a substance composed of a wide range of compounds. However, during the refining process of petroleum to obtain the raw material for polymer production, achieving absolute purity is challenging. Therefore, after purification processes, traces of substances that interfere as inhibitors of the ZN catalytic system may persist. Some of these molecules that have been studied, both theoretically and experimentally, as inhibitors of the ZN catalyst include formic acid, acetic acid, methanol, ethanol, isopropyl alcohol, 1-propanol, 1-butanol, 2-butanol, tert-butanol, carbon monoxide, carbon dioxide, molecular oxygen, iron oxide, carbon disulfide, hydrogen sulfide, arsine, carbonyl sulfide, ethyl mercaptan, methyl mercaptan, propyl mercaptan, butyl mercaptan, formaldehyde, propionaldehyde, butyraldehyde, furan, dimethylformamide, acetylene, and methylacetylene [8,19,20,21,22,23,24,25,26,27].

For this research, the Gaussian software version 16 was used. Density Functional Theory (DFT) has become essential in chemistry due to its accuracy and efficiency improvements since the 1990s. It offers a favorable cost/performance ratio compared to methods like Møller–Plesset perturbation theory and the coupled-cluster method, enabling the study of larger molecular systems. DFT is now the most widely used electronic structure method, crucial for modeling molecules, studying chemical reactions, and determining spectroscopic properties [28,29,30].

Although there are numerous studies on the Ziegler-Natta catalyst, few focus specifically on inhibitors and even fewer on the theoretical study of the reaction mechanisms by which inhibitors interact with the catalyst [23,30,31,32,33,34]. Therefore, this specific area related to the ZN catalyst requires more research to answer the fundamental questions that may arise. The present investigation aims to study the inhibition mechanism of acetylene and methylacetylene using DFT methods in the Gaussian 16 software, employing precise and reliable levels of theory. The goal is to generate results that allow for a clear understanding of how these species affect the Ziegler-Natta catalyst.

## 2. Results and Discussion

### 2.1. Optimization and Frequency of Species Involved

Optimization and frequency calculations were performed using the suggested methodology for ZN systems [3] (Section 3). These calculations yielded internal energy of −4.8 × 10^4^ and −7.3 × 10^4^ kcal mol^−1^ for acetylene and methylacetylene, respectively. Alkynes, such as acetylene and methylacetylene, are known for their low reactivity towards nucleophiles. However, their susceptibility to nucleophilic attacks can be enhanced when forming complexes with a metal center as a ligand. This increased susceptibility is attributed to the interaction of the alkyne with the metal center, resulting in a net displacement of electron density from the organic species towards the metal [35].

### 2.2. Inhibitor Molecular Reactivity

#### 2.2.1. Global Reactivity Descriptors

This section presents an analysis of the values obtained for the chemical descriptors of the species acetylene, methylacetylene, and propylene. The descriptors considered include the highest occupied molecular orbital energy (E_HOMO_), the lowest unoccupied molecular orbital energy (E_LUMO_), the chemical potential (μ), hardness (η), and electrophilicity (ω). These values are summarized in Table 1 and were obtained using the KID (Koopmans in DFT) procedure described in Section 3.3.1. The chemical potential (μ) was calculated as the additive inverse of electronegativity (χ).

The order of μ values for the species was $\mu_{ace}>\mu_{met}>\mu_{Pro}$, where $\mu_{ace}$, $\mu_{met}$, and $\mu_{Pro}$ are the chemical potential values for acetylene, methylacetylene, and propylene, respectively (see Table 2). This indicates that acetylene is the species with the lowest tendency to donate electrons. However, the difference in the chemical potential of acetylene is only 4.44% and 10% lower than that of methylacetylene and propylene, respectively. This difference can be explained by the hybridizations and the *s*-character of the bonds in these species. 

The sp hybridization in acetylene implies that the hybrid orbitals have a higher s-character (50%) compared to the sp^2^ hybridization in methylacetylene (33% s) and sp^3^ in propylene (25% s). This higher s-character in the sp orbitals means that the electrons are closer to the nucleus, resulting in a greater nuclear attraction and a lower tendency to donate electrons.

The same order of μ was observed for η in the species, with acetylene showing the highest hardness value. This suggests that acetylene is the most stable and least reactive species, followed by methylacetylene and propylene, respectively. These results are consistent with the μ values obtained and are justified by the same reasoning used for the chemical potential. In the case of ω, the trend changes, and methylacetylene has the highest electrophilicity value, followed by acetylene and propylene, respectively. This indicates that methylacetylene has the highest stabilization energy, meaning it releases the most energy to stabilize itself by accepting electron density with a maximum number of electrons. However, it is worth noting that, in general, in this study, alkynes exhibit higher electrophilicity than propylene.

#### 2.2.2. Local Reactivity Descriptors

**Figure 1:**
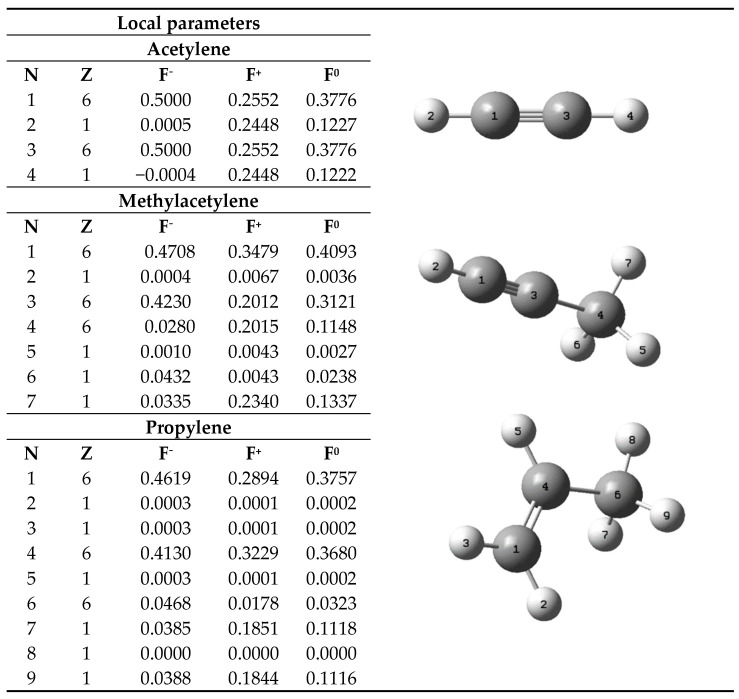
Numbered inhibitor and propylene molecules and reactivity values.

Reactivity studies were conducted using the UCA-FUKUI software to assess the reactive behavior of acetylene and methylacetylene as inhibitors, as well as propylene as the monomer in the first stage of polymerization, clarifying that the interaction of propylene with the metal was studied with the insertion of a methyl group into the ZN catalyst, originating from AlEt_3_. The results obtained are presented in Figure 1 and also show the atomic numbering assigned to each chemical species.

The results presented in Figure 1 provide a detailed description of the local reactivity parameters obtained through the UCA-FUKUI analysis, which examines each atom within the molecule. In this representation, N denotes a number used for atom enumeration in the molecule, while Z corresponds to the atomic number of each atom. The values of F^−^, F^+^, and F^0^ relate to the atom’s tendency to undergo a nucleophilic attack, its susceptibility to an electrophilic attack, and its susceptibility to a radical attack, respectively. For the acetylene molecule, the carbon atoms (atoms 1 and 3, as shown in Figure 1) exhibited the following order: F^−^ > F^0^ > F^+^, indicating a greater predisposition to nucleophilic attacks. It is noteworthy that the F^−^ values for both carbon atoms were 0.5, suggesting that the attack can be carried out by either of the two equivalently. On the other hand, the order F^0^ > F^+^ indicates that acetylene, under the studied conditions, is more susceptible to a radical attack than to an electrophilic attack. Compared to acetylene, methylacetylene will be analyzed, distinguishing between the non-methylated carbon (H–C≡), methylated carbon (≡C–CH_3_), and methyl group (–CH_3_). 

For methylacetylene, the order of the Fukui function parameters for atoms 1, 3, and 4—where 1 and 3 represent the alkyne carbons and 4 represents the alkane carbon—was as follows: F^−^ > F^0^ > F^+^, F^−^ > F^0^ > F^+^, and F^+^ > F^0^ > F^−^, respectively. When analyzing carbons 1 and 3 of methylacetylene, it was observed that, although the reactivity order remained consistent, there were variations in the values compared to acetylene, especially in carbon 3, which is bonded to the methyl group. In carbon 1, there was a 5.84% reduction in F^−^, a 26.64% increase in F^+^, and a 7.74% increase in F0. Meanwhile, in carbon 3, there was a 15.4% reduction in F^−^, a 21.15% reduction in F^+^, and a 17.34% reduction in F^0^. These percentages suggest that the presence of a methyl group in the acetylene molecule, equivalent to the methylacetylene molecule, results in a moderate loss of nucleophilic character and a significant increase in susceptibility to electrophilic and radical attacks for the non-methylated carbon, while a notable decrease in these three parameters is observed for the methylated carbon. In propylene, the carbon atoms are referenced with numbers 1, 4, and 6, with 1 and 4 having the alkene function and 6 having the alkane function. To compare propylene with acetylene and methylacetylene, we will discuss the non-methylated carbon, assigned to atom 1 in all three molecules; the methylated carbon, located at atom 3 in acetylene and methylacetylene, and at atom 4 in propylene; and the methyl group carbon, located at atom 4 in methylacetylene and atom 6 in propylene. For F^−^, F^+^, and F^0^, the results showed that for the non-methylated carbon of propylene, there is a 7.62% decrease and a 1.89% decrease compared to acetylene and methylacetylene, respectively, for F^−^, an 11.81% increase compared to acetylene and a 16.81% decrease compared to methylacetylene for F^+^, and a negligible variation for F^0^. For the methylated carbon of propylene, there is a significant 17.4% decrease and a slight 2.36% decrease compared to acetylene and methylacetylene, respectively, for F^−^, a 20.96% and 37.68% increase compared to acetylene and methylacetylene, respectively, for F^+^, and a 2.54% decrease compared to acetylene and a 15.19% increase compared to methylacetylene for F^0^.

The focus will be on the nucleophilic tendency of the molecules (F^−^), as this is the parameter involved in the reaction with the ZN catalyst, which consists of a catalytic center with a transition metal such as titanium. These results suggest that the tendency for a nucleophilic attack is higher for acetylene and methylacetylene than for propylene. This tendency varies by percentage from atom to atom, explaining a greater affinity of the metal center for alkynes compared to propylene. The analysis for the methyl group carbon will be omitted since, while it generates reactivity variations, it does not participate in the reactions of interest in this study.

### 2.3. Inhibitor Adsorption Energy

The adsorption energy of the inhibitors was evaluated by referencing two fundamental phases: the initial adsorption of ethyl from AlEt_3_ and the insertion of the first propylene molecule (see Supplementary Materials). In this context, the interaction involves the approach of a propylene molecule to the active site of the catalyst, forming a bond with the present ethyl radical. 

These phases were used as comparison points to determine the adsorption energy between two alternative inhibitors: acetylene and methylacetylene. To calculate this energy, the difference between the binding energy of the reaction adduct of acetylene or methylacetylene (adsorbate) with the Ziegler-Natta catalyst (adsorbent) and the sum of the energies of the isolated reactive species was evaluated. Specifically, in the case of ethyl insertion, the reactants considered were the Ziegler-Natta catalyst (TiCl_4_/MgCl_2_) and the co-catalyst (AlEt_3_), and the products were the ethyl group anchored to the ZN catalyst (ZnEt) and diethyl aluminum chloride (AlEt_2_Cl), which results from an atom of chlorine exchanged with the catalyst. For propylene, the energy associated with the incorporation of the first molecule into the polymer chain (chain creation) was recorded, subtracting the energy of this configuration of the adduct containing propylene from the energetic value of the individual reactive species involved. 

It is important to note that the insertion of the first propylene molecule acts as an intermediate in the overall process, as this insertion process occurs multiple times with several propylene molecules. All adsorption energies were calculated according to Equation (1), which is presented below:

(1) $$∆E=E_{Ads}=E_{Add}-\left(E_{Iso}+E_{ZN}\right)$$

where $E_{Ads}$ represents the adsorption energy, $E_{Add}$ is the energy of the adduct, $E_{Iso}$ is the energy of the isolated species, and $E_{ZN}$ is the energy of the canonical form of the catalyst (Figure 1). Table 3 presents the values obtained for acetylene, methylacetylene, ethyl, and propylene. In Table 1, it can be observed that for $E_{Ads}$, the value for ethyl is higher than that for acetylene and methylacetylene, indicating that the adducts of acetylene and methylacetylene achieve more stable adsorption. However, once ethyl has been adsorbed, the adsorption of propylene is even more stable than that of the inhibitors (2 kcal mol^−1^), suggesting that the inhibitors should be adsorbed before ethyl. Otherwise, the adsorption of propylene molecules would be stronger than that of the inhibitors. Barhi-Laleh et al. reported that the adsorption energies for propylene were −17.8 and −16.9 kcal mol^−1^ for propylene (1,2) and (2,1), respectively [3]. The value obtained for the absorption energy in the present study was 15.39 kcal mol^−1^. Taking the Barhi-Laleh value closest to the value obtained in this research as a reference, the error is calculated to be approximately 9%. This difference may be due to the different levels of theory used by each study, the size of the structure from which the calculations were performed, and/or the number of atoms considered in the structure.

**Figure 2:**
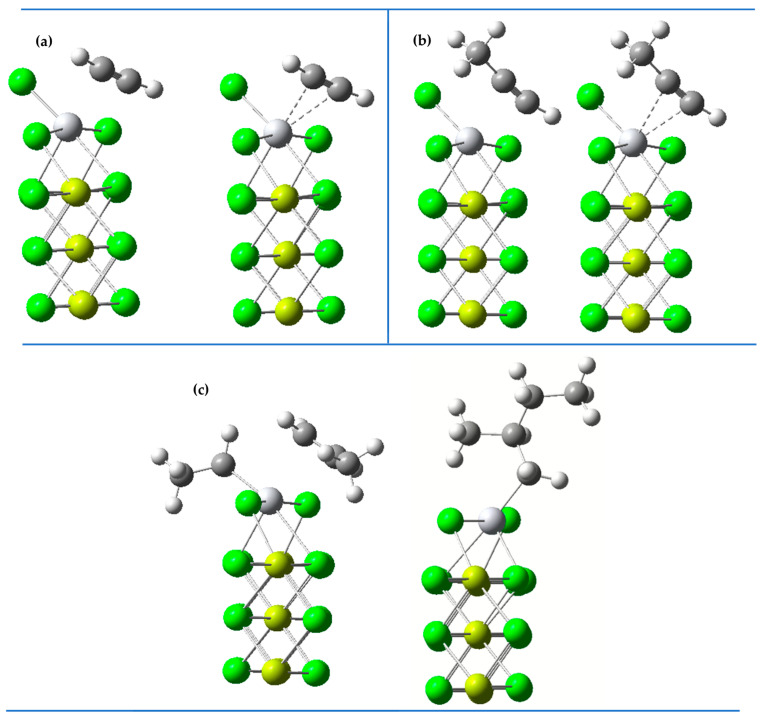
Reagents and products. (**a**) acetylene, (**b**) methylacetylene and (**c**) ethylene-propylene.

These results suggest that the interactions with the inhibitors are more energetically favorable, which could indicate that the catalytic centers have a preference for the inhibitors over propylene, provided the ethyl group has not been anchored beforehand. Figure 2 presents the geometric configurations of the reactants and products between the ZN catalyst, the inhibitors, and propylene. In Figure 2a–c, the structures on the left side of each pair represent the studied form of the reactants, while those on the right show the products, for acetylene, methylacetylene, and propylene, respectively.

### 2.4. Kinetic Constant and Inhibition Mechanisms

Equation (2), derived from Eyring’s transition state theory [36], was used to determine the velocity constants k:

(2) $$k=\frac{k_{b}T}{h}exp\left(-\frac{∆G^{‡}}{RT}\right)$$

The rate constants were calculated for acetylene, methylacetylene, and the initial polymerization of propylene, identified as k_act_, k_met_, and k_pro_, respectively (see Supplementary Materials). The order of magnitude of these constants was k_act_ > k_met_ > k_pro_. This finding suggests that, from a kinetic perspective, the inhibition reaction is more viable initially with acetylene, followed by methylacetylene, and lastly with propylene. Table 4 presents the values of the reactant energies (Ereac), the transition states (ETS), the change in free energy in the transition state (∆G^‡^), equivalent to the activation energy, and the rate constant (K) calculated from these parameters. The results obtained confirm that the reaction rates for the inhibition of acetylene and methylacetylene are considerably higher than those for propylene.

**Figure 3:**
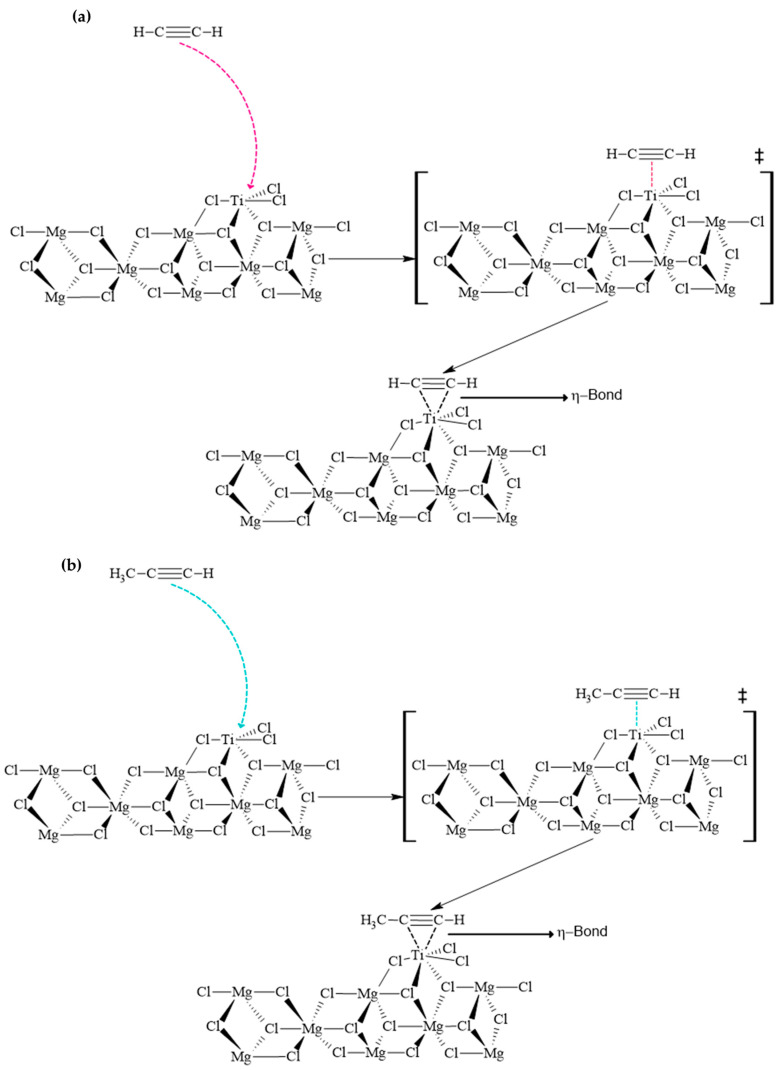
Reaction mechanisms for inhibitors and propylene. Structures with ‡ represents transition states. (**a**) depicts the approach of an acetylene molecule with the optimal orientation for an effective collision with the titanium metal center. (**b**) presents the reaction mechanism for methylacetylene, which is analogous to that of acetylene under similar conditions, with inherent differences due to the presence of an additional methyl group.

**Figure 4:**
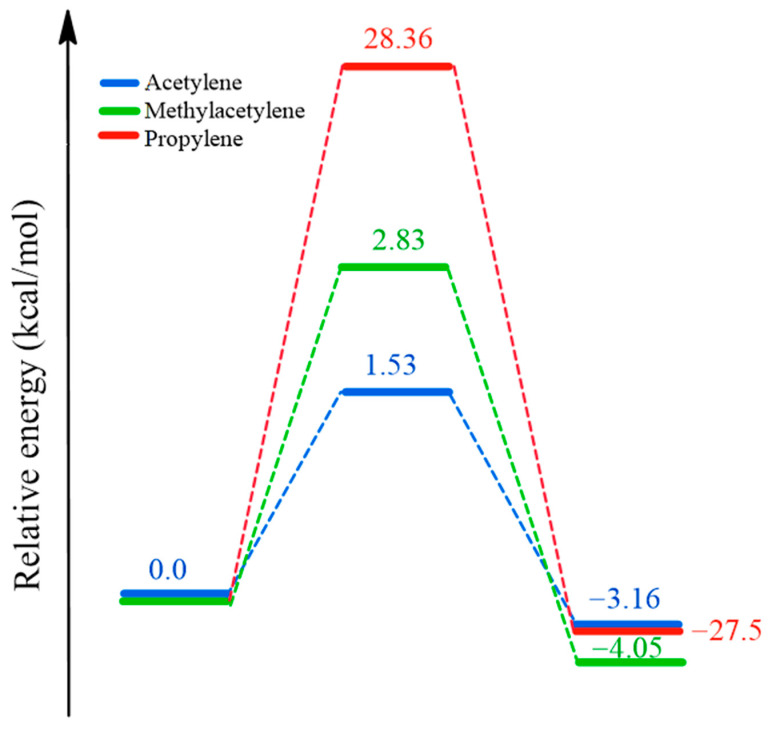
Reactivity profiles for inhibitors and propylene.

The transition states for acetylene and methylacetylene were obtained using a QST2 approach (see Supplementary Materials), due to the absence of significant geometric changes and considering the η-type bond interactions, which represent the reactive nature of alkynes with metal centers. This choice is based on the fact that in interactions involving η-bonds, there is a deformation of the bond (delocalization of π-electrons) but not necessarily the breaking and forming of a new bond. Therefore, although there is a change in geometry, it is not significant. In the acetylene and methylacetylene molecules, a metal-η-bond interaction is considered. Figure 3 illustrates the proposed mechanisms for the inhibition of acetylene and methylacetylene, along with their respective transition states and reactivity diagrams. Figure 3a depicts the approach of an acetylene molecule with the optimal orientation for an effective collision with the titanium metal center. Following this event, the interaction of the triple bond with the vacant d orbital of titanium occurs, where the triple bond deforms to partially overlap and transfer electron density to the metal’s d orbital. This overlapping phenomenon is represented in Figure 3a as the transition state. Subsequently, a maximum degree of overlap is reached, forming the η metal–alkyne bond. Figure 3b presents the reaction mechanism for methylacetylene, which is analogous to that of acetylene under similar conditions, with inherent differences due to the presence of an additional methyl group. On the other hand, Figure 4 details the reactivity profiles for the inhibitors and propylene, showing that the energy barrier propylene must overcome is 18.5 and 10.0 times greater than that of acetylene and methylacetylene, respectively.

These mechanisms, along with the values of the kinetic constants, adsorption energies (Section 2.2), and reactivity parameters (Section 2.1), justify the experimentally obtained results in a previous study [37]. Some nucleophiles present in the medium may react to form a new product; however, this topic is relevant to address in future research. Trace amounts between 0.03 and 40 ppm and 2 and 40 ppm, for acetylene and methylacetylene, respectively, generated notable effects in the polymerization process such as the decrease in catalyst productivity, which for this study are now understood as inhibition of active centers, the displacement (increase) of temperatures at which the first and second inflection points occur in the pyrolytic degradation of polypropylene produced with traces of these inhibitors, which already implies substantial changes in the properties of the produced polymer and changes in the mechanical properties of the polymer such as the melt flow index (MFI), which is related to the molecular weight of the polymer chain produced and consequently to the length of the chain. Flexural and tensile strength also decreased as a function of acetylene and methylacetylene concentration. All these properties are affected proportionally by the increase in acetylene and methylacetylene concentration [38].

### 2.5. HOMO, LUMO, and NBO Analysis for Chemical Species and Products

#### 2.5.1. HOMO and LUMO

In the course of this research, we found that Gaussian was unable to represent the HOMO and LUMO orbitals in the chemical reaction involving a crystalline network with an active center and a small molecule interacting with that center. This behavior can be attributed to several reasons inherent to the nature of the system and the capabilities of the software. First, the complexity of the system plays a significant role [3]. Molecular orbital calculations in crystalline networks, which include a large number of atoms and multiple complex interactions, are extremely computationally demanding [3,37]. This level of complexity may exceed the capabilities of Gaussian, which is optimized for simpler, discrete molecular systems. Moreover, the size of the system is a crucial factor. 

Crystalline networks require specific calculation methods that address the periodicity and extent of the system, such as band theory approaches. Gaussian, primarily designed for non-periodic molecular systems, may not be the most suitable software for these calculations, as its algorithms are not optimized for large-scale systems with periodic conditions. Another point to consider is the limitations of Gaussian’s algorithms. Calculations of individual molecular orbitals may not be accurate in large periodic systems due to the lack of algorithm optimization for these contexts [37,38]. 

The visualization of results presents its own difficulties. Handling and representing the data generated in large periodic system calculations can be technically challenging, and visualization tools may not effectively process the large amount of information involved [39].

**Figure 5:**
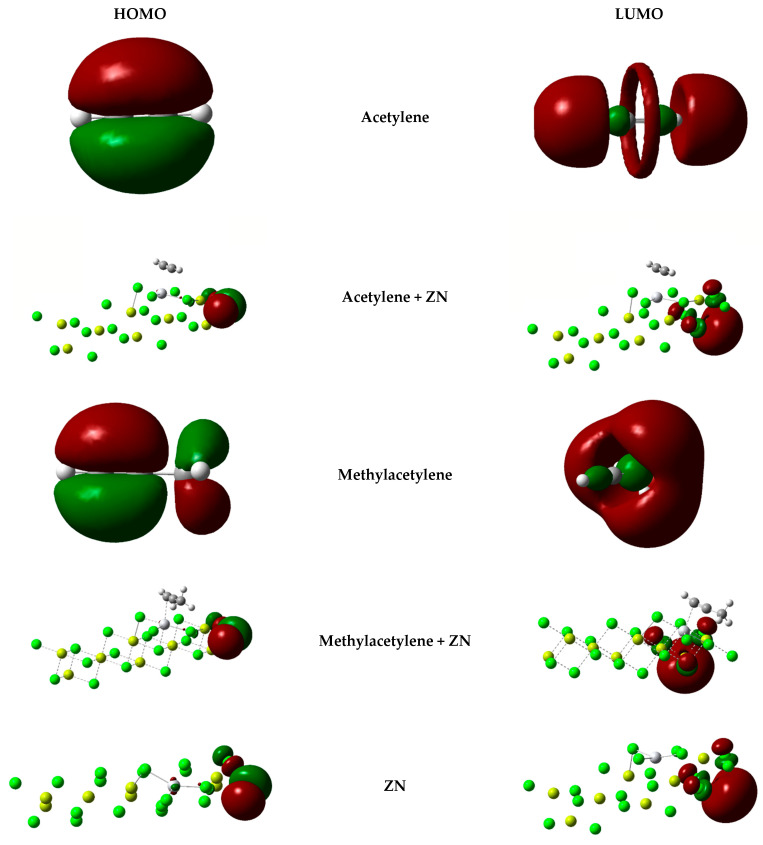
HOMO and LUMO orbitals for inhibitors and inhibitor products.

Finally, a supremely important point is the fact that the bond to be represented is not a common σ or π bond, but an η bond, which consequently generates images that do not reflect the interaction between the active titanium center and the inhibitors. However, Figure 5 shows the HOMO and LUMO of interest obtained in Gaussian.

#### 2.5.2. NBO

In the realm of computational chemistry, Natural Bond Orbitals (NBOs) stand as indispensable tools, providing a sophisticated framework for elucidating the electronic structure of molecules. Rooted in natural orbital theory, NBOs offer an intuitive representation of chemical bonding that surpasses traditional atomic or molecular orbitals [40,41]. Within the field of computational chemistry and the study of chemical reactions, NBOs serve several crucial purposes:

They enable detailed analyses of chemical bonds within molecules, identifying specific orbitals that contribute significantly to both covalent and non-covalent interactions, while quantifying phenomena such as van der Waals forces and resonance interactions [40,41].

NBOs provide valuable insights into molecular properties, offering data on partial charges, electron density distributions, and other relevant molecular characteristics. This information is crucial for predicting physical and chemical properties such as molecular polarity, chemical reactivity, and spectroscopic features [42,43].

Additionally, NBOs facilitate the study of charge transfer mechanisms within molecules or between molecules in intermolecular systems. This capability is crucial for understanding the dynamics of chemical reactions and for manipulating or controlling these processes [44,45,46].

Moreover, in the design of catalysts and functional materials, NBOs play a critical role in elucidating interactions between catalysts and substrates, thereby optimizing catalytic activity and reaction selectivity [47,48].

In the analysis of the NBO calculation results, a simple yet solid logic was adopted. Table 3 is based on the Gaussian output file for the NBO calculations, specifically on the values presented in the natural population analysis summary. The NBO calculations were performed for acetylene (ACT), methylacetylene (MET), the ZN catalyst focused on the titanium active center (Ti), and their products (ACT-ZN and MET-ZN).

Table 5 shows the reference atom, the number identifying the atom in the molecule (No), the charge distribution on the atom (Natural Charge), the valence of the atom in the molecule (Valence), the contribution of the valence electrons to higher diffuse orbitals (Rydberg), and the sum of these last three variables (Total). For titanium, it is noted that the natural charge values become more negative in the presence of an interaction with ACT and MET, indicating that titanium has gained a certain amount of charge. It is also observed that the average value for titanium is closer to the values for an interaction with the inhibitors. For the ACT molecule, the values of the natural charge, valence, Rydberg, and total for the carbon and hydrogen atoms decrease compared to the values of the product of this inhibitor (ACT-ZN). The natural charge decreases notably in the carbons, with a charge loss of 57.6% for each carbon atom compared to its charge in the isolated ACT molecule. 

In the case of MET, the change is more diverse due to the presence of the methyl group. Here, we observe that the carbon of the methyl group (C-No 1) has the highest natural charge, due to the electronegativity equalization effect from the hybridization of the atoms with a triple bond. The carbon bonded to the methyl group (C-No 2) acquires a negative charge, although considerably less than that of the other two carbons, as it is a secondary carbon and has two other carbon atoms attracting its electrons. The carbon not bonded to the methyl group (C-No 3) also has a negative natural charge, which is approximately one-third of the charge of the carbon with the highest charge. Upon examining the values for the MET-ZN product, it is noted that the carbon atom with the highest charge in the isolated MET molecule decreases its charge by 78.7%, and the charge of the carbon atom bonded to the methyl group becomes even positive. However, the carbon not bonded to the methyl group increases its charge by 24.2%, which does not match the percentage of charge loss of the carbon in the methyl group. This raises the question of whether, when the inhibitors interact with the titanium atom of the catalyst, there is merely a redistribution of the charge within the inhibitor molecule, or if there is a genuine transfer of electron density from the inhibitor to the titanium atom. From a simple inspection of the charge values, it is perceptible that the inhibitors are losing electron density. However, to clear any reasonable doubt, it is necessary to calculate the variation in natural charge, valence, and Rydberg for Ti in ZN compared to ACT-ZN and MET-ZN, and for ACT and MET compared to ACT-ZN and MET-ZN. For this purpose, a new variable called absolute sum (φ) is defined, which is calculated as follows:

(3) $$\varphi =\sum_{i}^{n}\left|C_{i}\right|\;Ec.$$

where (i) is the number identifying the atom in the molecule, n is the total number of atoms in the molecule, and C is the absolute value of the natural charge of the i-th atom in the molecule, then φ is simply the total natural charge of the molecule. Similar to thermodynamics, where it is determined if a reaction is exothermic or endothermic based on its sign, or in computational chemistry, where adsorption energy is calculated, it is possible to determine if a molecule loses or gains electron density in a reaction relative to the isolated molecule in the following way:

(4) $$∆\varphi =\varphi_{RM}-\varphi_{IM}\;Ec.$$

where $\varphi_{RM}$ is the total natural charge of the molecule in the reaction product and $\varphi_{RM}$ is the total natural charge of the isolated molecule. It follows logically that if ∆φ>0, meaning it is positive or approaches zero from the left or moves away from zero to the right relative to $\varphi_{IM}$, the molecule donates charge. Conversely, if ∆φ<0, meaning it is negative or approaches zero from the right or moves away from zero to the left relative to $\varphi_{IM}$, the molecule gains charge. This absolute sum can also be applied to a particular atom. This is another way to study donor-acceptor concepts.

Table 6 presents the values of φ for Ti, ACT, and MET, along with ∆φ, where ∆φ_ACT-Ti_ is the variation for ACT relative to ACT-ZN, ∆φ_Ti-ACT_ is the variation for MET relative to MET-ZN, ∆φ_Ti-ACT_ is the variation for Ti in ZN relative to ACT-ZN, and ∆φ_Ti-MET_ is the variation for Ti in ZN relative to MET-ZN. In Table 4, it can be observed that the variation values for the inhibitors are positive, and the values for titanium move away from zero towards the left relative to ∆φ_Ti_, which is equivalent to saying that ∆φ_Ti_ is negative. This indicates that, in the context of the reaction, the inhibitors donate electron density to the titanium center of the ZN catalyst. While the above may seem obvious, as it occurs in all chemical reactions and can be calculated through simpler and more familiar methods, Gaussian’s inability to graphically depict the involved HOMO and LUMO, due to the nature of the eta bond considered in the software where there is no bond breaking in the donor molecule, makes the method presented here a reliable analytical way to determine the presence or absence of bonding.

### 2.6. Prediction and Analysis of Theoretical IR Spectra

**Figure 6:**
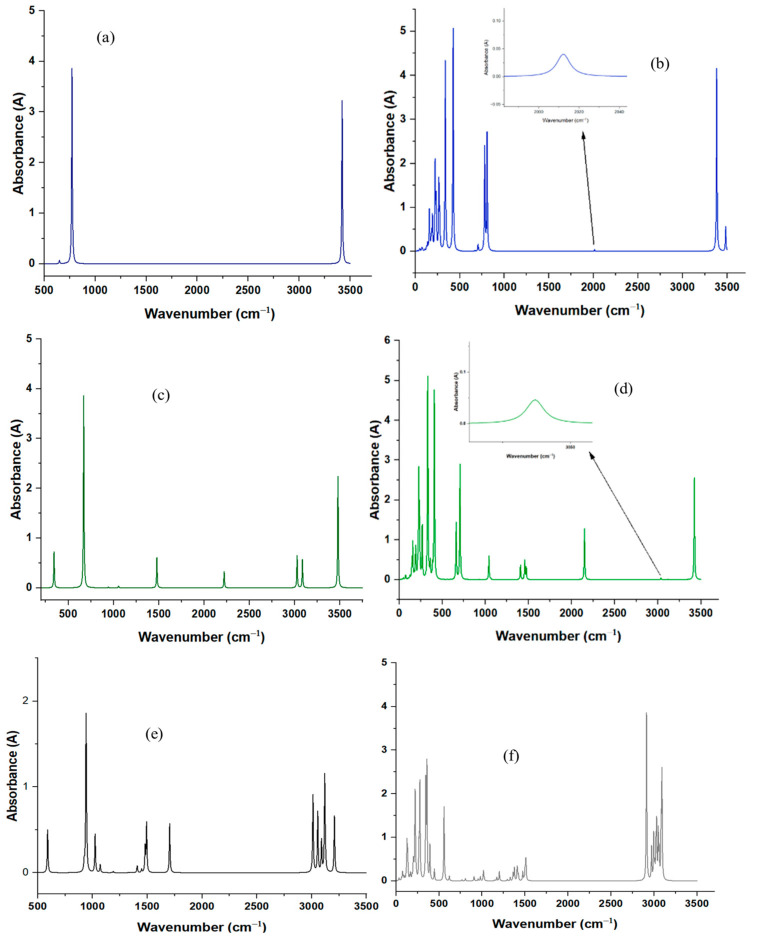
Calculated IR spectra for isolated species anchored to the catalytic center. (**a**) isolated acetylene and (**b**) its inhibition product, (**c**) isolated methylacetylene and (**d**) its inhibition product, (**e**) isolated propylene and (**f**) insertion of first propylene molecule.

From the frequency calculations, infrared (IR) spectra were generated for both isolated species and species anchored to the titanium active center in the ZN catalyst. Figure 6 shows these spectra, where Figure 6a,b correspond to acetylene and its anchored analog, Figure 6c,d represent methylacetylene and its anchored form, while Figure 6e,f reflect propylene and its first polymerization. 

On the other hand, Table 7 presents the theoretically obtained vibration frequencies, as well as the reported experimental values for the isolated species. The error of each peak was calculated compared to the nearest experimental value to the theoretical one obtained. Maximum errors of 2.7%, 5%, and 8.31%, and mean errors of 1.75%, 2.70%, and 1.52% were recorded for acetylene, methylacetylene, and propylene, respectively. These results suggest that the theoretical values obtained constitute an acceptable approximation of the actual vibration frequencies of each molecule.

Before this research, theoretical spectra of species bound to the metal center in the ZN catalyst (TiCl_4_/MgCl_2_) were not available. Therefore, these spectra represent a valuable starting point for future experimental investigations in infrared spectroscopy of this catalysis system interacting with the studied inhibitors. Given the previous results on frequency accuracy, we proceeded to compare the peaks between the isolated species and the species anchored to titanium in the catalytic center of the ZN catalyst, aiming to identify differences between the frequencies and characterize the interaction between the inhibitors and the ZN catalyst, as well as between propylene and the ZN catalyst. Table 8 presents this comparison between the anchored species and the isolated species. In this table, only the peaks of the anchored species found in the metal–alkyl interaction region are shown. In general, the number of frequencies obtained for the anchored species is much higher than for the isolated species, which can be corroborated by Figure 6. Additionally, for all anchored species, vibration modes between the titanium atom of the catalytic center and the species were recorded, at frequencies below 700 cm^−1^, specifically for acetylene 425 cm^−1^>, for methylacetylene 408 cm^−1^>, and for propylene 620 cm^−1^>. All frequencies above these values for each of the species correspond to vibrational modes of the species only, which do not represent vibrational interactions with the catalytic center. For acetylene, a total of 6 and 30 frequencies were obtained for the isolated and anchored species respectively. Of the 30 frequencies obtained for the anchored species, 23 were below the metal–alkyne interaction region (425 cm^−1^>), and of these 23, 16 corresponded to the metal–acetylene interaction. Similarly, for methylacetylene, 15 and 39 frequencies were obtained for the isolated and anchored species, respectively. Of the 39 frequencies for the anchored species, 26 were in the metal–alkyne interaction region (408 cm^−1^>), and of these 15 corresponded to metal–methylacetylene interactions. Likewise, for propylene, a total of 21 and 63 frequencies were recorded for the isolated and anchored species, respectively. Of the 63 frequencies for the anchored species, 29 were in the metal–alkene interaction region (620 cm^−1^>), and all corresponded to metal–propylene interactions. In Table 8, the frequencies corresponding to the interaction of each species with the metal are highlighted (in bold) and underlined to distinguish them from frequencies not corresponding to metal–alkyl interactions. These results suggest that for future experimental investigations using infrared spectroscopy, spectra should be obtained in the corresponding regions of mid-infrared and far-infrared, which are 4000–400 cm^−1^ and 400–10 cm^−1^, respectively. Ref. [49] in an experimental study observed several significant results in the study of Ziegler-Natta catalysts. The formation of Mg-Cl bonds was detected in all catalysts, with peaks observed in the range of 1633–1637 cm^−1^ and 2230–2260 cm^−1^. Additionally, Ti-Cl bands were identified in a range of 476–480 cm^−1^ and 608–618 cm^−1^ in all catalysts.

**Table 7:** Theoretical vs. experimental frequencies for acetylene, methylacetylene, and propylene.

	Theoretical Peaks	Experimental Peaks		
Species	Wavenumber (cm^−1^)	Wavenumber (cm^−1^)	Error %	Source
Acetylene	647.18	654/638/631/625	1.43	[35,50]
	649.52		1.80	
	772.43	780–730	0.97	
	2061.72	2066	0.20	
	3420.08	3339/3328/3305	0.23	
	3522.81	3428	2.7	
Methyl acetylene	343.34		4.99	[51]
	343.36	327/315/303	5.00	
	670.92	498	4.99	
	670.97	639/633	5.00	
	942.47	930/914/838	1.34	
	1055.99	1052/1048	0.37	
	1056	1115	0.38	
	1415.23	1452/1380	2.55	
	1478.55	2008	1.82	
	1478.56	2233	1.82	
	2221.52	2142/2110	0.14	
	3026.44	2616	1.52	
	3085.51	2981/2980/2931/2930	3.50	
	3085.53	3336/3334	3.50	
	3477.93		4.25	
Propylene	203.637		8.31	[52,53]
	590.285	188	2.43	
	923.222	576.27	0.42	
	941.008	912.67	0.57	
	948.278	919.29	1.34	
	1023.76	935.67	2.05	
	1070.22	990.77	2.39	
	1188.93	1045.20	1.61	
	1326.89	1170.04	2.23	
	1407.91	1297.86	0.85	
	1447.55	1377.94	0.33	
	1479.92	1420	2.57	
	1493.56	1442.71	3.52	
	1704.26	1653.18	3.08	
	3012.24	2931.46	0.09	
	3055.93	2954.30	1.13	
	3090.25	2973	0.02	
	3119.56	2991.03	0.92	
	3126.16	3015	1.13	
	3207.97	3091	3.78	

Ref. [54] conducted a theoretical study where they modeled the nano-sheets of the different faces (110, 104, and 107) of the ZN catalyst (50 MgCl_2_/TiCl_4_). Using DFT and machine learning techniques, they were able to predict IR spectra theoretically. In their analysis, they identified specific frequency values and ranges, including 157, 161, 100–200, 250, 263–267, 270, 275–280, 311, 322, 328, 340, 360, 382–398, 429, 435, 445, 436–459, and 493 cm^−^^1^. Significantly, they found that the obtained spectra were mainly located in the lower limit of the mid-infrared spectrum, with several peaks in the far-infrared region. These findings show notable consistency with the results obtained in the present study. Consequently, the importance of conducting additional experimental infrared studies on the ZN catalyst (TiCl_4_/MgCl_2_), particularly in the far-infrared region, is emphasized for a more comprehensive understanding of its behavior and properties.

## 3. Materials and Methods

### 3.1. Catalytic Center Prototype

**Figure 7:**
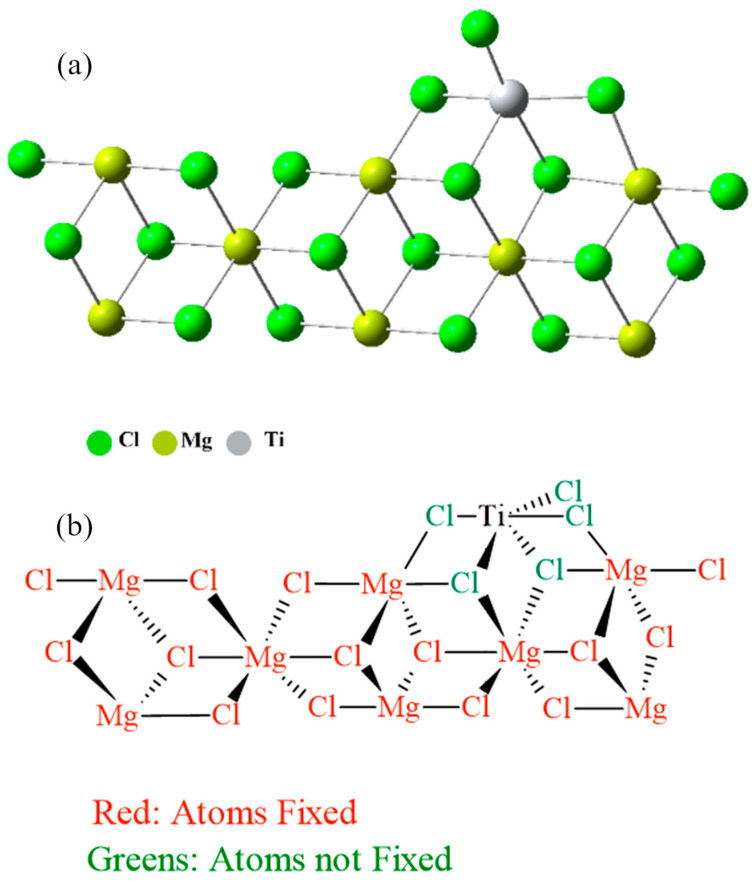
(**a**) Canonical form of the catalytic center and (**b**) Canonical form of the catalytic center with fixed atoms in red and free atoms in green and black.

The canonical configuration of the catalytic center was established (Figure 7a) based on previous studies conducted with other inhibitors, utilizing the 110 faces of MgCl_2_ crystals [55]. For these computations, a new model of the MgCl_2_ surface was utilized. In particular, the Mg_8_Cl_16_ cluster, which can be seen in Figure 7, was taken from relaxed MgCl_2_ surfaces. Based on previous calculations that showed poor or even unstable coordination of TiCl_4_ in the (104) plane, this model was selected. In contrast, TiCl_4_ has an energetically favorable coordination in the (110) plane [56]. These results contribute to our understanding of how the active Ti center on the MgCl_2_ surface is selectively impacted by impurity and how this interaction influences the catalytic process being studied. The inhibition mechanism of propylene polymerization was explored by freezing all atoms except for the titanium and the five chlorine atoms bonded to this metal. Figure 7b shows the atoms that were fixed to carry out the calculations.

### 3.2. Method, Functional, and Base Set

All calculations were performed using Gaussian 16 software. Calculations on methylacetylene and acetylene were carried out using DFT and the hybrid exchange-correlation functional combination developed by Becke, characterized by three parameters, and the correlation functional developed by Lee, Yang, and Parr, commonly known as B3LYP along with its D3 adjustment (B3LYP-D3) [57,58,59], together with the 6-311++G(d,p) basis set. For the study of the transition states (TS) of the inhibition mechanisms, the TS(Berny) and QST2 methods were selected, using the same levels of theory for DFT. Through combined optimization and frequency calculations for the catalytic center, acetylene, and methylacetylene, the optimal interaction distance was determined to be between 2.03 and 2.65 Å. All calculations were carried out under standard pressure and temperature conditions (1 atm and 298.15 K).

### 3.3. Reactivity of Species Involved

#### 3.3.1. Global Descriptors

For this study, calculations of geometric optimization and global reactivity descriptors for Ziegler-Natta catalyst inhibitors were performed. The calculations were carried out using the same level of theory described in Section 3.2, implemented in the Gaussian 16 software. The KID procedure was employed to calculate the global reactivity descriptors. This procedure involves the relationship of the frontier molecular orbital energies (HOMO and LUMO) with the ionization potential (I) and the electron affinity (A) of the system [60,61,62]. This method was used because it is valid for neutral and charged molecules [61]. The descriptors were calculated as shown in Table 9 below:

#### 3.3.2. Local Descriptors

In the methodological process used, a detailed examination was conducted on how the electron density changes in response to small variations, ρ(r), concerning the number of electrons, N, in the context of a given system and under the influence of a uniform and stable external potential, represented by υ(r). This derivative, expressed as ∂ρ(r)/∂N, is defined as the change in electron density concerning changes in the number of electrons and is calculated through the evaluation of the first derivative of the system’s total energy, E, pertaining to the electron density ρ(r). This analytical approach is fundamental for understanding molecular reactivity and for identifying regions with high and low electron density that can influence the susceptibility of a chemical species to participate in chemical reactions. This is calculated with the following equation:

(5) $$f\left(r\right)={\left[\frac{\partial \rho (r)}{\partial N}\right]}_{\nu (r)}={\left[\frac{\delta \mu }{\delta \nu (r)}\right]}_{N}$$

It is pertinent to highlight that the Fukui function provides a crucial tool for distinguishing susceptibilities to nucleophilic, electrophilic, and radical attacks. In the context of our study, single-point (SP) energy calculations were carried out using the DFT method, specifically with the B3LYP-D3 functional and the 6-311++G(d,p) basis set.

### 3.4. Calculation of Theoretical IR Spectra

Optimization and frequency calculations were carried out using the DFT method, with the B3LYP-D3 functional and the 6-311++G(d,p) basis set, to determine all the infrared (IR) spectra corresponding to the various species under study. These calculations allowed for the identification of significant changes in the IR spectra and regions of interest, thereby providing a solid foundation for future research involving experimentation and characterization through infrared spectroscopy.

## 4. Conclusions

The analyses conducted in this study provide a profound insight into the interaction between inhibitors and the catalyst in the polymerization process, which has significant implications for the efficiency and selectivity of this industrial process. Optimization calculations and reactivity studies were performed using acetylene and methylacetylene as inhibitors, and propylene as a reference monomer. The results revealed a higher tendency of the inhibitors to undergo nucleophilic attacks compared to propylene. Additionally, the presence of a methyl group in methylacetylene increased its susceptibility to electrophilic and radical attacks, with reductions of F^−^ of 15.4% and 17.34% for carbon 1 and 3, respectively. The adsorption energy of the inhibitors, compared to that of ethyl (a crucial precursor in the polymerization process), revealed greater stability in the adsorption of inhibitors compared to ethyl. Adsorption energy values of −11.10 and −13.99 kcal mol^−1^ were obtained for acetylene and methylacetylene respectively. Calculations of the rate constants for inhibition with acetylene, methylacetylene, and propylene suggest that the inhibition reaction is kinetically more feasible with acetylene, followed by methylacetylene and propylene. Values of 4.68 × 10^11^, 5.29 × 10^10^, and 5.29 × 10^−^^8^ M^−^^1^ s^−^^1^ were obtained. The proposed mechanisms suggest a direct interaction between inhibitors and the catalytic center through triple bond deformation and the formation of an η interaction. The activation energy was determined using the Eyring equation, with ΔG^‡^ values of 1.53, 2.83, and 28.36 kcal mol^−^^1^ for acetylene, methylacetylene, and propylene respectively. Additionally, theoretical infrared spectra obtained for isolated species and anchored to the ZN catalyst showed an acceptable approximation of theoretical frequencies to actual ones. Specific frequencies indicating interactions between inhibitors and the catalytic center were identified.

## Figures and Tables

**Table 1:** Structures of different Ziegler-Natta catalysts.

Structural Formula ZN	Molecular Formula ZN	Reference
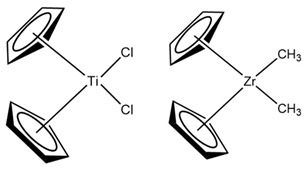	[Cp_2_Zr(CH_3_)_2_] [Cp_2_Ti(Cl)_2_]	[14]
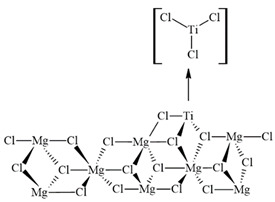	[TiCl_3_]	[15]
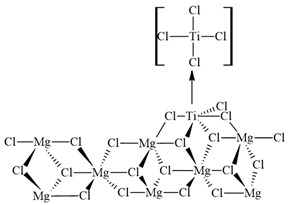	[TiCl_4_]	[17]

**Table 2:** Chemical potential, hardness, and electrophilicity values for inhibitors and propylene.

Chemical Species	Descriptor Value				
	E_HOMO_	E_LUMO_	µ	η	ω
Acetylene	−0.30139	0.00393	−0.14873	0.30532	0.03622
Methylacetylene	−0.27599	−0.00825	−0.14212	0.26774	0.03771
Propylene	−0.26288	−0.00466	−0.13377	0.25822	0.03464

**Table 3:** Adsorption energies and energy variation of inhibitors and Propylene.

Thermodynamic Properties					
Compound/E (kcal mol^−1^)	E_Iso_	Dipole Moment (D)	$E_{Iso}+E_{ZN}$	E_Add_	∆E
Acetylene	−48,541.12	0.0009	−7,074,222.17	−7,074,233.28	−11.10
Methylacetylene	−73,225.12	0.8525	−7,098,906.16	−7,098,920.15	−13.99
Ethyl	−301,331.2	0.1315	−7,327,012.26	−7,327,012.57	−0.31
Propylene (First polymerization)	−74,009.27	0.4023	−6,860,579.58	−6,860,594,97	−15.39

**Table 4:** Parameters for kinetic constant calculations and their values.

Compound/E (kcal mol^−1^)	E_reac_	E_TS_	$∆G^{‡}$	K
Acetylene	−7,074,243.32	−7,074,241.78	1.53	4.65 × 10^11^
Methylacetylene	−7,098,914.13	−7,098,911.31	2.82	5.29 × 10^10^
Propylene (First polymerization)	−6,786,568,35	−6,860,508.34	28.36	2.3 × 10^−8^

**Table 5:** General NBO population analysis. Values in bold are the average of the parameters for titanium and the absolute sum and totals of the parameters for acetylene, methylacetylene and their inhibition products.

Atom	No.	Natural Charge	Valence	Rydberg	Total
Ti(ZN)	25	−0.34951	2.17486	0.17678	11.34951
Ti(ACT-ZN)	25	−0.46227	2.22382	0.24007	11.46227
Ti(MET-ZN)	25	−0.44502	2.20543	0.24149	11.44502
**Average**	**-**	**−0.41893**	**2.20137**	**0.21944667**	**11.4189333**
C(ACT)	1	−0.22205	4.21468	0.00858	6.22205
C(ACT)	2	−0.22205	4.21468	0.00858	6.22205
H(ACT)	3	0.22205	0.77598	0.00197	0.77795
H(ACT)	4	0.22205	0.77598	0.00197	0.77795
**TOTAL**	**-**	**0**	**9.98132**	**0.0211**	**14**
φ		**0.8882**			
C(ACT-ZN)	29	−0.09246	2.08523	0.00796	3.09246
C(ACT-ZN)	30	−0.09406	2.08696	0.00783	3.09406
H(ACT-ZN)	31	0.13652	0.36080	0.00268	0.36348
H(ACT-ZN)	32	0.13581	0.36190	0.00229	0.36419
**TOTAL**	**-**	**0**	**4.89489**	**0.02076**	**6.91419**
φ		**0.45885**			
C(MET)	1	−0.65046	4.64338	0.00786	6.65046
C(MET)	2	−0.00652	3.99703	0.01073	6.00652
C(MET)	3	−0.24977	4.24556	0.00572	6.24977
H(MET)	4	0.22748	0.77036	0.00216	0.77252
H(MET)	5	0.22748	0.77036	0.00216	0.77252
H(MET)	6	0.22749	0.77036	0.00216	0.77251
H(MET)	7	0.22431	0.77374	0.00195	0.77569
**TOTAL**	**-**	**0**	**15.97079**	**0.03274**	**21.99999**
φ		**1.81351**			
C(MET-ZN)	29	−0.13862	2.13189	0.00761	3.13862
C(MET-ZN)	31	0.04179	1.94997	0.00898	2.95821
C(MET-ZN)	32	−0.32989	2.32434	0.00595	3.32989
H(MET-ZN)	30	0.13719	0.35993	0.00288	0.36281
H(MET-ZN)	33	0.12918	0.36942	0.00140	0.37082
H(MET-ZN)	34	0.12498	0.37413	0.00089	0.37502
H(MET-ZN)	35	0.12577	0.37286	0.00136	0.37423
**TOTAL**	**-**	**0**	**7.88254**	**0.02907**	**10.9096**
φ		**1.02742**			

**Table 6:** Variations of NBO parameters for titanium, acetylene, and methylacetylene.

Parameter	φ_Ti_	φ_ACT_	φ_MET_	∆φ_ACT-Ti_	∆φ_MET-Ti_	∆φ_Ti-ACT_	∆φ_Ti-MET_
Natural Charge	−0.34951	0.8882	1.81351	0.42935	0.78609	−0.11276	−0.09551

**Table 8:** Comparison of infrared frequencies for inhibitors and their products with the ZN catalyzer. frequencies corresponding to the interaction of each species with the metal are highlighted (in bold) and underlined to distinguish them from frequencies not corresponding to metal–alkyl interactions.

Peak Comparison					
Acetylene + ZN	Acetylene	Methyl Acetylene + ZN	Methyl Acetylene	Propylene + ZN	Propylene
Wavenumber (cm^−1^)	Wavenumber (cm^−1^)	Wavenumber (cm^−1^)	Wavenumber (cm^−1^)	Wavenumber (cm^−1^)	Wavenumber (cm^−1^)
23.16	647.18	20.38	343.34	**12.73**	203.637
48.74	649.52	**37.14**	343.36	**23.54**	590.285
53.69	772.43	49.20	670.92	**33.71**	923.222
73.26	2061.72	70.41	670.97	**49.16**	941.008
**80.70**	3420.08	74.69	942.47	**58.56**	948.278
**82.43**	3522.81	76.83	1055.99	**63.19**	1023.76
**107.97**		99.50	1056	**74.71**	1070.22
**120.47**		114.82	1415.23	**86.45**	1188.93
**126.58**		122.58	1478.55	**99.08**	1326.89
**138.46**		125.21	1478.56	**117.57**	1407.91
**158.50**		136.74	2221.52	**126.98**	1447.55
**172.16**		**145.77**	3026.44	**134.93**	1479.92
**177.30**		**156.95**	3085.51	**158.71**	1493.56
**181.87**		**173.99**	3085.53	**167.32**	1704.26
**191.47**		**177.02**	3477.93	**185.32**	3012.24
195.15		**189.84**		**199.58**	3055.93
**222.84**		**193.16**		**205.42**	3090.25
231.92		**214.80**		**220.54**	3119.56
235.33		**227.34**		**224.09**	3126.16
**265.07**		**235.07**		**237.88**	3207.97
**274.99**		**239.08**		**265.78**	
**337.33**		**331.60**		**270.81**	
**424.00**		**359.25**		**276.99**	
		**386.96**		**345.24**	
		**407.51**		**358.60**	
				**393.57**	
				**445.71**	
				**557.98**	
				**619.35**	

**Table 9:** Expressions for the calculation of global descriptors.

Property or Descriptor	Symbol	Equation Based on HOMO and LUMO
Electronegativity	χ	$\chi =-\frac{1}{2}(I+A)\approx \frac{1}{2}(\varepsilon_{L}+\varepsilon_{H})$
Chemical Potential	µ	μ=−χ
Globlal Hardness	η	$\eta =I-A\approx \varepsilon_{L}-\varepsilon_{H}$
Electrophilicity	ω	$\omega =\frac{\chi^{2}}{2\eta }$

Where $\varepsilon_{H}$ and $\varepsilon_{L}$ are the energies of the HOMO and LUMO orbitals, respectively.

## Data Availability

Data are contained within the article.

## Supplementary Materials

The following supporting information can be downloaded at: https://www.mdpi.com/article/10.3390/ijms251910585/s1.
