# Supplementary material for: Computational Study on the Inhibition Mechanisms of the Ziegler-Natta Catalyst in the Propylene Polymerization Process: Part 1 Effects of Acetylene and Methylacetylene
Source: Int J Mol Sci. 2024 Oct 1;25(19):10585. doi: 10.3390/ijms251910585 (PMC11476528; doi:10.3390/ijms251910585)
Supplement: Supplementary file 1 [file ijms-25-10585-s001.zip › ijms-3092185-supplementary.pdf]

Supporting Information

Coordinates:

Chemical Species:

Acetylene:

Input orientation:

Center Number	Atomic Number	Atomic Type	Coordinates (Angstroms) X	Y	Z
1	6	0	11.735773	-6.664338	-0.155612
2	6	0	10.569773	-6.667760	0.125121
3	1	0	12.765551	-6.661486	-0.403404
4	1	0	9.540002	-6.670616	0.372895

SCF Done: E = -77.3566392698 a.u. after 7 cycles

Methylacetylene:

Input orientation:

Center Number	Atomic Number	Atomic Type	Coordinates (Angstroms) X	Y	Z
1	6	0	16.561200	-6.445300	-0.117600
2	6	0	15.248400	-7.021700	-0.334100
3	6	0	14.152900	-7.502700	-0.514800
4	1	0	16.453400	-5.450600	0.365600
5	1	0	17.155000	-7.114500	0.541300
6	1	0	17.079500	-6.328600	-1.093500
7	1	0	13.147000	-7.944300	-0.680800

SCF Done: E = -116.690210166 a.u. after 7 cycles

Propylene:

Input orientation:

Center Number	Atomic Number	Atomic Type	Coordinates (Angstroms) X	Y	Z
1	6	0	-2.782178	-0.445545	0.000000
2	1	0	-2.249015	-1.373249	0.000000
3	1	0	-3.852178	-0.445545	0.000000
4	6	0	-2.106904	0.729433	0.000000
5	1	0	-2.640068	1.657138	0.000000
6	6	0	-0.566904	0.729433	0.000000
7	1	0	-0.210238	0.223337	-0.872672
8	1	0	-0.210238	1.738237	-0.001956
9	1	0	-0.210237	0.226725	0.874628

SCF Done: E = -117.938784189 A.U. after 11 cycles

ZN Catalyst ($TiCl_4/MgCl_2$):

Input orientation:

Center Number	Atomic Number	Atomic Type	Coordinates (Angstroms) X	Y	Z
1	17	0	-7.659939	-1.285699	0.942298

2	12	0	-6.630520	-3.100912	-0.444483
3	17	0	-8.537192	0.664582	-1.755454
4	12	0	-6.488833	0.535147	-0.318432
5	17	0	-4.582162	-3.230348	0.992538
6	17	0	-1.362661	-1.538957	1.168834
7	17	0	-4.440475	0.405711	1.118589
8	17	0	-2.381639	-3.224714	-1.654977
9	12	0	-0.333341	-3.354138	-0.217994
10	17	0	-5.459416	-1.280066	-1.705215
11	12	0	-3.411058	-1.409499	-0.268191
12	17	0	-2.239945	0.411332	-1.528936
13	12	0	-0.191523	0.281898	-0.091744
14	17	0	1.715004	-3.483537	1.219068
15	17	0	4.934509	-1.792113	1.395482
16	17	0	1.787954	0.017773	1.727702
17	17	0	3.915689	-3.477959	-1.428372
18	12	0	5.963961	-3.607389	0.008579
19	17	0	0.837903	-1.533315	-1.478657
20	12	0	2.886252	-1.662719	-0.041941
21	17	0	4.010391	0.283628	-1.139824
22	12	0	6.105454	0.028557	0.134598
23	17	0	8.154016	-0.100762	1.571633
24	17	0	7.135066	-1.786520	-1.252183
25	22	0	2.484285	2.368146	1.676085
26	17	0	4.758595	2.350394	1.507396
27	17	0	0.851955	2.545060	-0.107016
28	17	0	1.623067	3.478122	3.385790

SCF Done: E= -11196.2436832   A.U. after   30 cycles

Trimethylaluminum:

Input orientation:

Center Number	Atomic Number	Atomic Type	Coordinates (Angstroms) X	Y	Z
1	13	0	-0.161905	0.238095	0.000000
2	6	0	1.858095	0.238095	0.000000
3	1	0	2.214762	1.110626	0.506338
4	1	0	2.214762	-0.636672	0.502465
5	6	0	-1.171905	-1.511273	-0.003392
6	1	0	-2.223209	-1.316068	-0.042811
7	1	0	-0.883905	-2.087738	-0.857584
8	6	0	-1.171905	1.987463	0.003392
9	1	0	-1.962795	1.944080	-0.715990
10	1	0	-1.583432	2.159825	0.975933
11	6	0	-0.211323	3.135506	-0.358381
12	1	0	-0.762448	4.048878	-0.441522
13	1	0	0.530094	3.234687	0.406710
14	1	0	0.265804	2.920618	-1.291694
15	6	0	2.371428	0.241313	-1.451923
16	1	0	2.224159	1.210766	-1.880144
17	1	0	3.414173	0.001566	-1.462090
18	1	0	1.832618	-0.486156	-2.022336
19	6	0	-0.843317	-2.296219	1.280153
20	1	0	-1.525597	-3.114083	1.382585
21	1	0	0.157100	-2.671382	1.222546
22	1	0	-0.933149	-1.648578	2.127142

SCF Done: E = -480.192807807 a.u. after 7 cycles

Diethyl aluminum chloride:

Input orientation:

Center Number	Atomic Number	Atomic Type	Coordinates (Angstroms) X	Y	Z
1	13	0	-0.195330	0.424263	0.105666
2	6	0	-1.262152	-1.236806	0.302792
3	1	0	-2.121475	-1.014751	0.951060
4	1	0	-1.717794	-1.444510	-0.677686
5	6	0	-1.185532	2.126060	-0.135916
6	1	0	-1.907819	1.976497	-0.951830
7	1	0	-1.819048	2.264499	0.753036
8	6	0	-0.368401	3.407143	-0.392470
9	1	0	-1.007088	4.289632	-0.510208
10	1	0	0.322238	3.618297	0.430036
11	1	0	0.237770	3.326658	-1.300451
12	6	0	-0.539520	-2.501209	0.808282
13	1	0	-1.207934	-3.367886	0.859881
14	1	0	0.296093	-2.778242	0.158153
15	1	0	-0.124970	-2.357425	1.811332
16	17	0	2.043444	0.350203	0.108518

SCF Done: E = -861.257653254 a.u. after 7 cycles

Reagent Optimization and Frequencies (Chemical Species + ZN Catalyst):

Acetylene + ZN Catalyst:

Input orientation (Distance 3.0 Angstroms):

Center Number	Atomic Number	Atomic Type	Coordinates (Angstroms) X	Y	Z
1	17	0	-0.982301	-0.539823	0.000000
2	12	0	0.047118	-2.355036	-1.386781
3	17	0	-1.859554	1.410458	-2.697752
4	12	0	0.188805	1.281023	-1.260730
5	17	0	2.095476	-2.484472	0.050240
6	17	0	5.311463	-0.791047	0.226167
7	17	0	2.237163	1.151587	0.176291
8	17	0	4.295999	-2.478838	-2.597275
9	12	0	6.344199	-2.608371	-1.160555
10	17	0	1.218222	-0.534190	-2.647513
11	12	0	3.266529	-0.663533	-1.210408
12	17	0	4.437442	1.157525	-2.470845
13	12	0	6.493585	1.022459	-1.032412
14	17	0	8.392668	-2.737556	0.276955
15	17	0	11.612179	-1.046284	0.453284
16	17	0	8.543309	0.996218	0.459454
17	17	0	10.593472	-2.732187	-2.370426
18	12	0	12.641672	-2.861506	-0.933677
19	17	0	7.515242	-0.787642	-2.421400
20	12	0	9.562849	-0.914320	-0.985533
21	17	0	10.676877	1.038706	-2.232574
22	12	0	12.780357	0.774930	-0.808064
23	17	0	14.831652	0.645210	0.629349
24	17	0	13.812962	-1.040533	-2.194431
25	22	0	9.629077	3.014701	-0.717482

26	17	0	11.764251	2.674207	0.393718
27	17	0	7.600788	2.908823	-2.093660
28	17	0	8.847147	4.548948	0.679727
29	6	0	11.316989	5.123026	-2.023591
30	6	0	10.587572	4.738868	-2.906517
31	1	0	11.992808	5.510161	-1.295164
32	1	0	9.983792	4.470312	-3.743637

SCF Done: E = -11273.6184603 A.U. after 35 cycles

Input orientation (Distance 2.62 Angstroms):

Center Number	Atomic Number	Atomic Type	Coordinates (Angstroms) X	Y	Z
1	17	0	-0.982301	-0.539823	0.000000
2	12	0	0.047118	-2.355036	-1.386781
3	17	0	-1.859554	1.410458	-2.697752
4	12	0	0.188805	1.281023	-1.260730
5	17	0	2.095476	-2.484472	0.050240
6	17	0	5.311389	-0.791058	0.226079
7	17	0	2.237163	1.151587	0.176291
8	17	0	4.295999	-2.478838	-2.597275
9	12	0	6.344251	-2.608380	-1.160518
10	17	0	1.218222	-0.534190	-2.647513
11	12	0	3.266541	-0.663553	-1.210426
12	17	0	4.437444	1.157520	-2.470849
13	12	0	6.493585	1.022534	-1.032251
14	17	0	8.392659	-2.737549	0.276957
15	17	0	11.612152	-1.046249	0.453304

16	17	0	8.489362	0.883510	0.624107
17	17	0	10.593475	-2.732187	-2.370425
18	12	0	12.641671	-2.861510	-0.933674
19	17	0	7.515230	-0.787647	-2.421423
20	12	0	9.562875	-0.914361	-0.985564
21	17	0	10.617209	1.053284	-2.109240
22	12	0	12.780390	0.774911	-0.808154
23	17	0	14.831641	0.645213	0.629364
24	17	0	13.812965	-1.040543	-2.194416
25	22	0	9.600270	2.854748	-0.327793
26	17	0	11.905394	2.927387	0.167234
27	17	0	7.703944	2.958427	-1.851259
28	17	0	8.915099	4.517206	0.950656
29	6	0	10.295596	4.419363	-2.379721
30	6	0	9.129496	4.416057	-2.099104
31	1	0	11.329144	4.421884	-2.628520
32	1	0	8.095947	4.412688	-1.850327

SCF Done: E= -11273.4816525 A.U. after 33 cycles

Methylacetylene + ZN Catalyst:

Input orientation (Distance 3.0 Angstroms):

Center Number	Atomic Number	Atomic Type	Coordinates (Angstroms) X	Y	Z
1	17	0	0.293542	-0.303327	0.000000
2	12	0	1.322961	-2.118540	-1.386781
3	17	0	-0.583711	1.646954	-2.697752

4	12	0	1.464648	1.517519	-1.260730
5	17	0	3.371319	-2.247976	0.050240
6	17	0	6.590692	-0.556539	0.226483
7	17	0	3.513006	1.388083	0.176291
8	17	0	5.571842	-2.242342	-2.597275
9	12	0	7.620157	-2.371769	-1.160281
10	17	0	2.494065	-0.297694	-2.647513
11	12	0	4.542427	-0.427134	-1.210496
12	17	0	5.713527	1.393685	-2.471223
13	12	0	7.762194	1.264329	-1.033951
14	17	0	9.668482	-2.501151	0.276787
15	17	0	12.887996	-0.809701	0.453188
16	17	0	9.810783	1.212426	0.468013
17	17	0	11.869175	-2.495580	-2.370675
18	12	0	13.917434	-2.625021	-0.933720
19	17	0	8.791357	-0.550937	-2.420939
20	12	0	10.839768	-0.680593	-0.984326
21	17	0	11.962663	1.275480	-2.221373
22	12	0	14.058798	1.011047	-0.807699
23	17	0	16.107500	0.881603	0.629331
24	17	0	15.088554	-0.804149	-2.194475
25	22	0	10.901016	3.259329	-0.681597
26	17	0	13.019443	2.892756	0.421923
27	17	0	8.883949	3.149684	-2.077123
28	17	0	10.040496	4.743443	0.748738
29	6	0	11.866481	4.911487	-2.679331
30	1	0	11.423938	4.495349	-3.555989
31	6	0	12.447367	5.558553	-1.831538
32	6	0	13.170974	6.416275	-0.907091

33	1	0	13.932533	5.850982	-0.366795
34	1	0	13.656759	7.213336	-1.477443
35	1	0	12.487217	6.863044	-0.182255

SCF Done: E = -11312.9606966 A.U. after 36 cycles

Input orientation (Distance 2.62 Angstroms):

Center Number	Atomic Number	Atomic Type	Coordinates (Angstroms) X	Y	Z
1	17	0	0.293542	-0.303327	0.000000
2	12	0	1.322961	-2.118540	-1.386781
3	17	0	-0.583711	1.646954	-2.697752
4	12	0	1.464648	1.517519	-1.260730
5	17	0	3.371319	-2.247976	0.050240
6	17	0	6.590820	-0.556585	0.226536
7	17	0	3.513006	1.388083	0.176291
8	17	0	5.571842	-2.242342	-2.597275
9	12	0	7.620140	-2.371766	-1.160292
10	17	0	2.494065	-0.297694	-2.647513
11	12	0	4.542423	-0.427127	-1.210489
12	17	0	5.713536	1.393704	-2.471234
13	12	0	7.761958	1.264270	-1.034042
14	17	0	9.668485	-2.501165	0.276770
15	17	0	12.887990	-0.809741	0.453184
16	17	0	9.741435	1.000145	0.785404
17	17	0	11.869170	-2.495587	-2.370670
18	12	0	13.917442	-2.625017	-0.933719
19	17	0	8.791384	-0.550943	-2.420955

20	12	0	10.839733	-0.680347	-0.984239
21	17	0	11.963872	1.266000	-2.082122
22	12	0	14.058935	1.010929	-0.807700
23	17	0	16.107497	0.881610	0.629335
24	17	0	15.088547	-0.804148	-2.194481
25	22	0	10.437766	3.350518	0.733787
26	17	0	12.712076	3.332766	0.565098
27	17	0	8.805436	3.527432	-1.049314
28	17	0	9.576548	4.460494	2.443492
29	6	0	10.470673	5.687126	-0.160744
30	1	0	9.400673	5.687126	-0.160744
31	6	0	11.671873	5.687126	-0.160744
32	6	0	13.211873	5.687126	-0.160744
33	1	0	13.568540	5.213521	0.729979
34	1	0	13.568540	5.152541	-1.016260
35	1	0	13.568540	6.695317	-0.195951

SCF Done: E = -11312.8957785 A.U. after 32 cycles

Propylene + ZN Catalyst

Input orientation:

Center Number	Atomic Number	Atomic Type	Coordinates (Angstroms) X	Y	Z
1	17	0	-2.339090	-2.064364	0.000000
2	12	0	-1.309670	-3.879577	-1.386781
3	17	0	-3.216343	-0.114083	-2.697752
4	12	0	-1.167983	-0.243518	-1.260730
5	17	0	0.738688	-4.009013	0.050240

6	*17*	*0*	*3.954600*	*-2.315599*	*0.226079*
7	*17*	*0*	*0.880375*	*-0.372954*	*0.176291*
8	*17*	*0*	*2.939211*	*-4.003379*	*-2.597275*
9	*12*	*0*	*4.987463*	*-4.132921*	*-1.160517*
10	*17*	*0*	*-0.138566*	*-2.058731*	*-2.647513*
11	*12*	*0*	*1.909752*	*-2.188094*	*-1.210427*
12	*17*	*0*	*3.080656*	*-0.367021*	*-2.470850*
13	*12*	*0*	*5.136781*	*-0.501994*	*-1.032253*
14	*17*	*0*	*7.035864*	*-4.262088*	*0.276955*
15	*17*	*0*	*10.255362*	*-2.570785*	*0.453309*
16	*17*	*0*	*7.148591*	*-0.605914*	*0.518888*
17	*17*	*0*	*9.236680*	*-4.256727*	*-2.370431*
18	*12*	*0*	*11.284881*	*-4.386053*	*-0.933672*
19	*17*	*0*	*6.158438*	*-2.312186*	*-2.421414*
20	*12*	*0*	*8.206142*	*-2.438923*	*-0.985567*
21	*17*	*0*	*9.352309*	*-0.553193*	*-2.211185*
22	*12*	*0*	*11.423579*	*-0.749633*	*-0.808154*
23	*17*	*0*	*13.474853*	*-0.879328*	*0.629363*
24	*17*	*0*	*12.456178*	*-2.565082*	*-2.194417*
25	*22*	*0*	*8.231071*	*1.524362*	*-0.470250*
26	*17*	*0*	*10.469698*	*1.316318*	*0.297906*
27	*17*	*0*	*6.183066*	*1.545595*	*-1.832215*
28	*6*	*0*	*9.153842*	*3.132233*	*-2.604605*
29	*1*	*0*	*8.392398*	*2.789345*	*-3.301757*
30	*6*	*0*	*8.762339*	*3.832242*	*-1.522075*
31	*1*	*0*	*7.723586*	*4.112236*	*-1.385472*
32	*1*	*0*	*9.482551*	*4.255643*	*-0.829781*
33	*6*	*0*	*10.573193*	*2.879259*	*-3.005265*
34	*1*	*0*	*10.798806*	*3.488771*	*-3.888463*

35	1	0	11.279339	3.142419	-2.216879
36	1	0	10.724763	1.838861	-3.301990
37	6	0	7.507059	2.677596	1.154361
38	1	0	7.762478	3.733838	1.009982
39	1	0	6.415292	2.606954	1.032296
40	6	0	7.908576	2.229444	2.566565
41	1	0	8.982838	2.341724	2.732578
42	1	0	7.396194	2.827165	3.330584
43	1	0	7.656687	1.183015	2.756601

SCF Done: E = -10933.1512854 A.U. after 39 cycles

Reaction products:

Acetylene + ZN Catalyst:

Input orientation:

Center Number	Atomic Number	Atomic Type	Coordinates (Angstroms) X	Y	Z
1	17	0	-0.982301	-0.539823	0.000000
2	12	0	0.047118	-2.355036	-1.386781
3	17	0	-1.859554	1.410458	-2.697752
4	12	0	0.188805	1.281023	-1.260730
5	17	0	2.095476	-2.484472	0.050240
6	17	0	5.311463	-0.791047	0.226167
7	17	0	2.237163	1.151587	0.176291
8	17	0	4.296000	-2.478838	-2.597275
9	12	0	6.344199	-2.608372	-1.160556
10	17	0	1.218222	-0.534190	-2.647513
11	12	0	3.266529	-0.663533	-1.210408

12	*17*	*0*	*4.437442*	*1.157525*	*-2.470845*
13	*12*	*0*	*6.493586*	*1.022459*	*-1.032412*
14	*17*	*0*	*8.392669*	*-2.737556*	*0.276955*
15	*17*	*0*	*11.612180*	*-1.046284*	*0.453284*
16	*17*	*0*	*8.543309*	*0.996219*	*0.459454*
17	*17*	*0*	*10.593473*	*-2.732188*	*-2.370426*
18	*12*	*0*	*12.641673*	*-2.861507*	*-0.933677*
19	*17*	*0*	*7.515243*	*-0.787642*	*-2.421400*
20	*12*	*0*	*9.562850*	*-0.914321*	*-0.985533*
21	*17*	*0*	*10.676878*	*1.038706*	*-2.232574*
22	*12*	*0*	*12.780358*	*0.774930*	*-0.808064*
23	*17*	*0*	*14.831653*	*0.645210*	*0.629349*
24	*17*	*0*	*13.812963*	*-1.040533*	*-2.194431*
25	*22*	*0*	*9.629078*	*3.014702*	*-0.717482*
26	*17*	*0*	*11.764252*	*2.674207*	*0.393718*
27	*17*	*0*	*7.600789*	*2.908823*	*-2.093660*
28	*17*	*0*	*8.847148*	*4.548949*	*0.679727*
29	*6*	*0*	*11.055622*	*4.796560*	*-1.821344*
30	*6*	*0*	*10.326205*	*4.412401*	*-2.704270*
31	*1*	*0*	*11.731441*	*5.183694*	*-1.092917*
32	*1*	*0*	*9.722425*	*4.143845*	*-3.541390*

SCF Done: E = -11273.6246380 a.u. after 13 cycles

Methylacetylene + ZN Catalyst:

Input orientation:

Center Number	Atomic Number	Atomic Type	Coordinates (Angstroms) X	Y	Z

1	17	0	1.064736	0.724020	0.000000
2	12	0	2.094155	-1.091193	-1.386781
3	17	0	0.187483	2.674302	-2.697752
4	12	0	2.235842	2.544867	-1.260730
5	17	0	4.142513	-1.220629	0.050240
6	17	0	7.361888	0.470807	0.226483
7	17	0	4.284200	2.415431	0.176291
8	17	0	6.343036	-1.214995	-2.597275
9	12	0	8.391352	-1.344422	-1.160281
10	17	0	3.265259	0.729653	-2.647513
11	12	0	5.313622	0.600213	-1.210496
12	17	0	6.484721	2.421032	-2.471223
13	12	0	8.533385	2.291675	-1.033952
14	17	0	10.439676	-1.473804	0.276786
15	17	0	13.659191	0.217645	0.453188
16	17	0	10.582030	2.239842	0.467968
17	17	0	12.640370	-1.468233	-2.370675
18	12	0	14.688629	-1.597673	-0.933720
19	17	0	9.562551	0.476410	-2.420939
20	12	0	11.610962	0.346758	-0.984326
21	17	0	12.733850	2.302846	-2.221423
22	12	0	14.829995	2.038393	-0.807699
23	17	0	16.878695	1.908951	0.629331
24	17	0	15.859749	0.223199	-2.194475
25	22	0	11.672175	4.286776	-0.681601
26	17	0	13.790771	3.920250	0.421794
27	17	0	9.655220	4.177003	-2.077277
28	17	0	10.811628	5.770824	0.748744
29	6	0	12.443755	5.650462	-2.535154

30	1	0	12.001088	5.234161	-3.411672
31	6	0	13.024686	6.297650	-1.687494
32	6	0	13.748399	7.155439	-0.763170
33	1	0	14.509707	6.590051	-0.222634
34	1	0	14.234472	7.952228	-1.333644
35	1	0	13.064657	7.602563	-0.038540

SCF Done : E = -11312.9660799 A.U. after 33 cycles

Propylene + ZN Catalyst:

Input orientation:

Center Number	Atomic Number	Atomic Type	Coordinates (Angstroms) X	Y	Z
1	17	0	-1.058694	0.860540	-7.775776
2	12	0	0.420984	-0.743366	-9.006996
3	17	0	-0.294125	3.547657	-9.787211
4	12	0	0.707260	2.634963	-7.679657
5	17	0	1.422368	-1.656060	-6.899445
6	17	0	4.187023	-0.791551	-4.697191
7	17	0	1.708644	1.722269	-5.572106
8	17	0	4.668000	-1.485540	-8.034551
9	12	0	5.669428	-2.398254	-5.927286
10	17	0	2.186937	1.031058	-8.910881
11	12	0	3.188263	0.118422	-6.803276
12	17	0	4.953890	1.893022	-6.706927
13	12	0	5.960764	0.973072	-4.596147
14	17	0	6.670627	-3.310805	-3.819265
15	17	0	9.437973	-2.449176	-1.615573

16	17	0	6.870657	0.148667	-2.364570
17	17	0	9.916429	-3.140631	-4.954226
18	12	0	10.917798	-4.053131	-2.846946
19	17	0	7.435324	-0.623852	-5.831417
20	12	0	8.436956	-1.533643	-3.724311
21	17	0	10.185801	0.370938	-3.631585
22	12	0	11.201833	-0.673639	-1.521308
23	17	0	12.205434	-1.587390	0.587931
24	17	0	12.683955	-2.278642	-2.750660
25	22	0	8.594722	1.990610	-2.240521
26	17	0	9.895526	1.078159	-0.402147
27	17	0	7.471586	2.818748	-4.267844
28	6	0	9.658577	4.507343	-1.103318
29	1	0	10.267352	4.043522	-0.316115
30	6	0	8.321224	3.742712	-1.175667
31	1	0	7.922531	3.480566	-0.185454
32	1	0	7.560078	4.315876	-1.725845
33	6	0	10.424479	4.357000	-2.433793
34	1	0	10.642584	3.300492	-2.686400
35	1	0	9.873002	4.795193	-3.273715
36	1	0	11.409067	4.836285	-2.386376
37	6	0	9.484879	5.999008	-0.750329
38	1	0	8.879517	6.481742	-1.531274
39	1	0	10.473974	6.478302	-0.787022
40	6	0	8.854358	6.249979	0.622422
41	1	0	7.837354	5.846546	0.680080
42	1	0	9.444894	5.783409	1.420437
43	1	0	8.796603	7.323106	0.835177

SCF Done: E = -10932.0963022   A.U. after   40 cycles

States of transition

Acetylene + ZN Catalyst:

Input orientation:

Center Number	Atomic Number	Atomic Type	Coordinates (Angstroms) X	Y	Z
1	17	0	-0.982301	-0.539823	0.000000
2	12	0	0.047118	-2.355036	-1.386781
3	17	0	-1.859554	1.410458	-2.697752
4	12	0	0.188805	1.281023	-1.260730
5	17	0	2.095476	-2.484472	0.050240
6	17	0	5.311463	-0.791047	0.226167
7	17	0	2.237163	1.151587	0.176291
8	17	0	4.296000	-2.478838	-2.597275
9	12	0	6.344199	-2.608372	-1.160556
10	17	0	1.218222	-0.534190	-2.647513
11	12	0	3.266529	-0.663533	-1.210408
12	17	0	4.437442	1.157525	-2.470845
13	12	0	6.493586	1.022459	-1.032412
14	17	0	8.392669	-2.737556	0.276955
15	17	0	11.612180	-1.046284	0.453284
16	17	0	8.542936	0.975101	0.472561
17	17	0	10.593473	-2.732188	-2.370426
18	12	0	12.641673	-2.861507	-0.933677
19	17	0	7.515243	-0.787642	-2.421400
20	12	0	9.562850	-0.914321	-0.985533
21	17	0	10.683494	1.037149	-2.230083

22	12	0	12.780358	0.774930	-0.808064
23	17	0	14.831653	0.645210	0.629349
24	17	0	13.812963	-1.040533	-2.194431
25	22	0	9.618914	3.006068	-0.689351
26	17	0	11.750113	2.685313	0.388705
27	17	0	7.623772	2.895722	-2.100468
28	17	0	8.754246	4.498925	0.725493
29	6	0	10.874114	4.949005	-1.699461
30	6	0	10.414200	4.364883	-2.651510
31	1	0	11.279870	5.533645	-0.905265
32	1	0	10.069147	3.906908	-3.550830

SCF Done: E = -11273.6239665   A.U. after   22 cycles

Methylacetylene + ZN Catalyst:

Input orientation:

Center Number	Atomic Number	Atomic Type	Coordinates (Angstroms) X	Y	Z
1	17	0	0.293542	-0.303327	0.000000
2	12	0	1.322961	-2.118540	-1.386781
3	17	0	-0.583711	1.646954	-2.697752
4	12	0	1.464648	1.517519	-1.260730
5	17	0	3.371319	-2.247976	0.050240
6	17	0	6.590692	-0.556539	0.226483
7	17	0	3.513006	1.388083	0.176291
8	17	0	5.571842	-2.242342	-2.597275
9	12	0	7.620158	-2.371770	-1.160281
10	17	0	2.494065	-0.297694	-2.647513

11	*12*	*0*	*4.542428*	*-0.427134*	*-1.210496*
12	*17*	*0*	*5.713527*	*1.393685*	*-2.471223*
13	*12*	*0*	*7.762194*	*1.264329*	*-1.033951*
14	*17*	*0*	*9.668483*	*-2.501151*	*0.276787*
15	*17*	*0*	*12.887997*	*-0.809701*	*0.453188*
16	*17*	*0*	*9.923626*	*1.340166*	*0.456704*
17	*17*	*0*	*11.869176*	*-2.495580*	*-2.370675*
18	*12*	*0*	*13.917435*	*-2.625021*	*-0.933720*
19	*17*	*0*	*8.791357*	*-0.550937*	*-2.420939*
20	*12*	*0*	*10.839769*	*-0.680593*	*-0.984327*
21	*17*	*0*	*12.114598*	*1.444280*	*-2.250964*
22	*12*	*0*	*14.058799*	*1.011047*	*-0.807699*
23	*17*	*0*	*16.107501*	*0.881603*	*0.629331*
24	*17*	*0*	*15.088555*	*-0.804149*	*-2.194475*
25	*22*	*0*	*11.047328*	*3.408897*	*-0.690876*
26	*17*	*0*	*13.147112*	*3.010000*	*0.432007*
27	*17*	*0*	*9.070456*	*3.380981*	*-2.141416*
28	*17*	*0*	*10.183898*	*4.885029*	*0.745969*
29	*6*	*0*	*11.819389*	*4.755061*	*-2.566884*
30	*1*	*0*	*11.382767*	*4.354143*	*-3.453545*
31	*6*	*0*	*12.378483*	*5.358477*	*-1.689004*
32	*6*	*0*	*13.097909*	*6.198659*	*-0.745345*
33	*1*	*0*	*13.848025*	*5.620429*	*-0.202687*
34	*1*	*0*	*13.597388*	*6.998340*	*-1.299983*
35	*1*	*0*	*12.409032*	*6.642474*	*-0.023523*

SCF Done: E= -11312.9571246   A.U. after   32 cycles

Propylene + ZN Catalyst:

Input orientation:

Center Number	Atomic Number	Atomic Type	Coordinates (Angstroms) X	Y	Z
1	17	0	-1.912200	-2.913042	-0.587688
2	12	0	-0.882780	-4.728256	-1.974470
3	17	0	-2.789453	-0.962761	-3.285441
4	12	0	-0.741093	-1.092196	-1.848419
5	17	0	1.165578	-4.857692	-0.537448
6	17	0	4.381490	-3.164277	-0.361609
7	17	0	1.307265	-1.221632	-0.411397
8	17	0	3.366101	-4.852058	-3.184964
9	12	0	5.414353	-4.981600	-1.748206
10	17	0	0.288324	-2.907409	-3.235202
11	12	0	2.336642	-3.036772	-1.798116
12	17	0	3.507546	-1.215699	-3.058539
13	12	0	5.563671	-1.350672	-1.619942
14	17	0	7.462754	-5.110767	-0.310733
15	17	0	10.682253	-3.419463	-0.134379
16	17	0	7.602190	-1.417698	-0.140802
17	17	0	9.663570	-5.105406	-2.958120
18	12	0	11.711772	-5.234732	-1.521361
19	17	0	6.585328	-3.160864	-3.009103
20	12	0	8.633032	-3.287601	-1.573256
21	17	0	9.715797	-1.372262	-2.809401
22	12	0	11.850470	-1.598311	-1.395843
23	17	0	13.901744	-1.728006	0.041675
24	17	0	12.883069	-3.413760	-2.782106
25	22	0	8.720377	0.676702	-1.348922

26	*17*	*0*	*11.104981*	*0.566493*	*-0.560099*
27	*17*	*0*	*6.550617*	*0.653688*	*-2.567942*
28	*6*	*0*	*9.456457*	*3.110463*	*-2.580199*
29	*1*	*0*	*10.229556*	*3.412989*	*-1.877689*
30	*6*	*0*	*9.672894*	*2.006207*	*-3.334710*
31	*1*	*0*	*10.617666*	*1.479916*	*-3.289442*
32	*1*	*0*	*8.980848*	*1.721727*	*-4.121069*
33	*6*	*0*	*8.303799*	*4.048675*	*-2.778633*
34	*1*	*0*	*8.676823*	*4.961278*	*-3.258951*
35	*1*	*0*	*7.530270*	*3.620325*	*-3.417790*
36	*1*	*0*	*7.857010*	*4.356817*	*-1.830811*
37	*6*	*0*	*8.177600*	*2.061418*	*0.189232*
38	*1*	*0*	*9.098283*	*1.496339*	*0.329963*
39	*1*	*0*	*7.521650*	*2.032661*	*-0.681380*
40	*6*	*0*	*7.752577*	*2.960620*	*1.299061*
41	*1*	*0*	*7.789673*	*4.019749*	*1.007820*
42	*1*	*0*	*6.723152*	*2.756427*	*1.613006*
43	*1*	*0*	*8.403228*	*2.837739*	*2.165614*

SCF Done: E = -10933.1170992 A.U. after 34 cycles

Reaction pathways

IRC

Acetylene + ZN Catalyst:

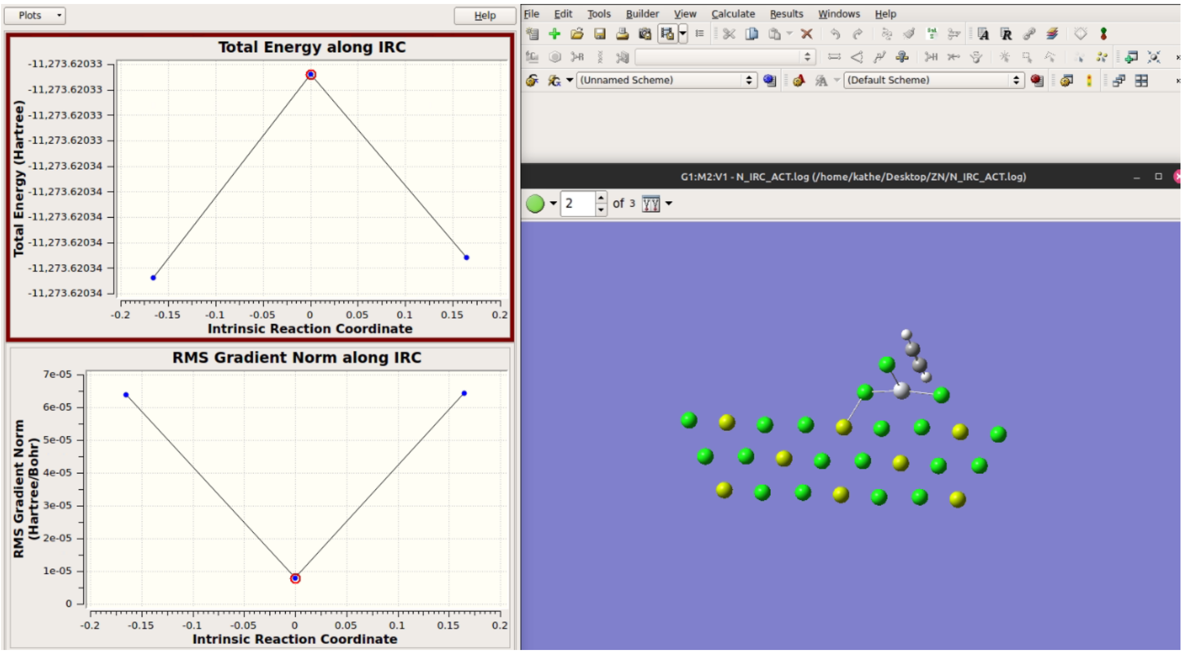

Input orientation:

Center Number	Atomic Number	Atomic Type	Coordinates (Angstroms) X	Y	Z
1	17	0	-0.982301	-0.539823	0.000000
2	12	0	0.047118	-2.355036	-1.386781
3	17	0	-1.859554	1.410458	-2.697752
4	12	0	0.188805	1.281023	-1.260730
5	17	0	2.095476	-2.484472	0.050240
6	17	0	5.311463	-0.791047	0.226167
7	17	0	2.237163	1.151587	0.176291
8	17	0	4.296000	-2.478838	-2.597275
9	12	0	6.344199	-2.608372	-1.160556
10	17	0	1.218222	-0.534190	-2.647513
11	12	0	3.266529	-0.663533	-1.210408
12	17	0	4.437442	1.157525	-2.470845
13	12	0	6.493586	1.022459	-1.032412

14	*17*	*0*	*8.392669*	*-2.737556*	*0.276955*
15	*17*	*0*	*11.612180*	*-1.046284*	*0.453284*
16	*17*	*0*	*8.532167*	*0.963589*	*0.467901*
17	*17*	*0*	*10.593473*	*-2.732188*	*-2.370426*
18	*12*	*0*	*12.641673*	*-2.861507*	*-0.933677*
19	*17*	*0*	*7.515243*	*-0.787642*	*-2.421400*
20	*12*	*0*	*9.562850*	*-0.914321*	*-0.985533*
21	*17*	*0*	*10.697827*	*1.022144*	*-2.230880*
22	*12*	*0*	*12.780358*	*0.774930*	*-0.808064*
23	*17*	*0*	*14.831653*	*0.645210*	*0.629349*
24	*17*	*0*	*13.812963*	*-1.040533*	*-2.194431*
25	*22*	*0*	*9.622768*	*3.004743*	*-0.657899*
26	*17*	*0*	*11.766199*	*2.759490*	*0.326203*
27	*17*	*0*	*7.650040*	*2.917860*	*-2.057664*
28	*17*	*0*	*8.822630*	*4.413652*	*0.942498*
29	*6*	*0*	*10.198675*	*5.231558*	*-1.746263*
30	*6*	*0*	*10.584255*	*4.383496*	*-2.515473*
31	*1*	*0*	*9.877669*	*6.017616*	*-1.101153*
32	*1*	*0*	*10.947913*	*3.725939*	*-3.272402*

SCF Done: E = -11272.8536885   A.U. after   33 cycles

Methylacetylene + <u>ZN Catalyst</u>:

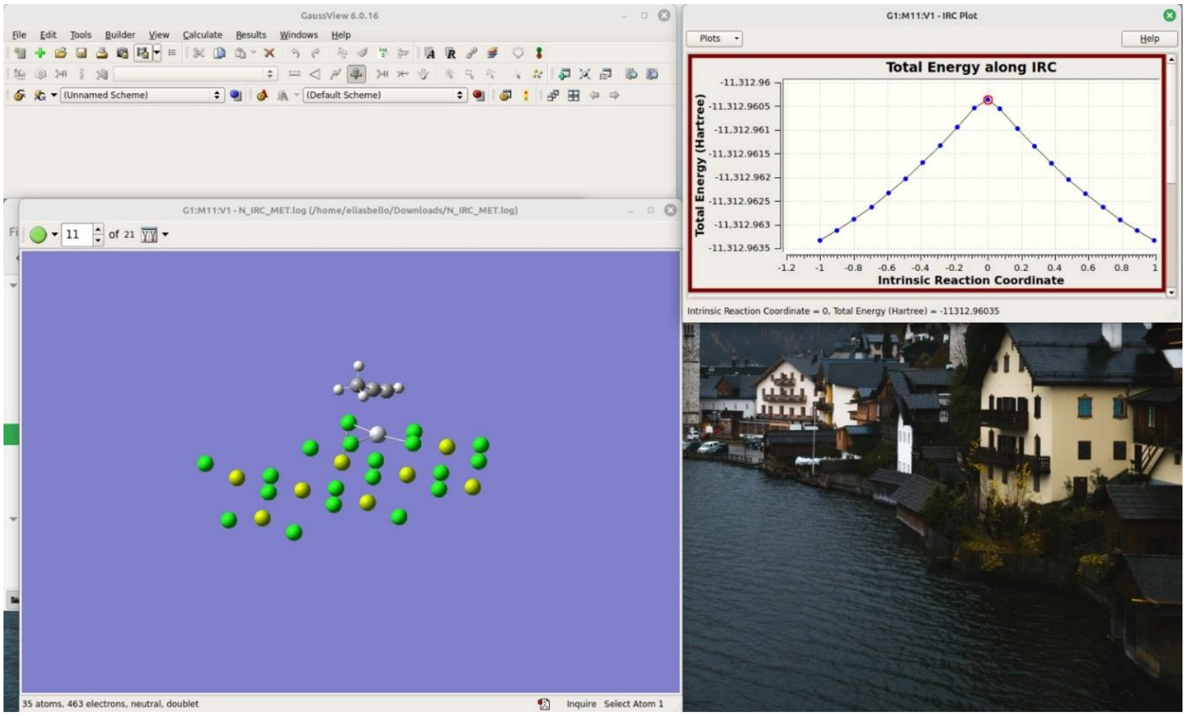

IRC-IRC-IRC-IRC-IRC-IRC-IRC-IRC-IRC-IRC-IRC-IRC-IRC-IRC-IRC-IRC-IRC-IRC

Input orientation:

Center Number	Atomic Number	Atomic Type	Coordinates (Angstroms) X	Y	Z
1	17	0	0.293542	-0.303327	0.000000
2	12	0	1.322961	-2.118540	-1.386781
3	17	0	-0.583711	1.646954	-2.697752
4	12	0	1.464648	1.517519	-1.260730
5	17	0	3.371319	-2.247976	0.050240
6	17	0	6.590692	-0.556539	0.226483
7	17	0	3.513006	1.388083	0.176291
8	17	0	5.571842	-2.242342	-2.597275
9	12	0	7.620158	-2.371770	-1.160281

10	*17*	*0*	*2.494065*	*-0.297694*	*-2.647513*
11	*12*	*0*	*4.542428*	*-0.427134*	*-1.210496*
12	*17*	*0*	*5.713527*	*1.393685*	*-2.471223*
13	*12*	*0*	*7.762194*	*1.264329*	*-1.033951*
14	*17*	*0*	*9.668483*	*-2.501151*	*0.276787*
15	*17*	*0*	*12.887997*	*-0.809701*	*0.453188*
16	*17*	*0*	*9.802779*	*1.193476*	*0.465868*
17	*17*	*0*	*11.869176*	*-2.495580*	*-2.370675*
18	*12*	*0*	*13.917435*	*-2.625021*	*-0.933720*
19	*17*	*0*	*8.791357*	*-0.550937*	*-2.420939*
20	*12*	*0*	*10.839769*	*-0.680593*	*-0.984327*
21	*17*	*0*	*11.973305*	*1.245067*	*-2.226165*
22	*12*	*0*	*14.058799*	*1.011047*	*-0.807699*
23	*17*	*0*	*16.107501*	*0.881603*	*0.629331*
24	*17*	*0*	*15.088555*	*-0.804149*	*-2.194475*
25	*22*	*0*	*10.893854*	*3.247360*	*-0.644813*
26	*17*	*0*	*13.047503*	*2.998400*	*0.326269*
27	*17*	*0*	*8.911366*	*3.173065*	*-2.038230*
28	*17*	*0*	*10.100038*	*4.625425*	*0.982728*
29	*6*	*0*	*11.813769*	*4.610037*	*-2.450007*
30	*1*	*0*	*12.124770*	*3.894362*	*-3.177077*
31	*6*	*0*	*11.535893*	*5.601928*	*-1.808312*
32	*6*	*0*	*11.258636*	*6.860876*	*-1.136932*
33	*1*	*0*	*11.762668*	*6.909743*	*-0.170607*
34	*1*	*0*	*11.623901*	*7.674201*	*-1.771511*
35	*1*	*0*	*10.187771*	*6.990380*	*-0.974129*

SCF Done: E = -11312.9603532   A.U. after   39 cycles
